# Green Supplier Selection Using Fuzzy Multiple-Criteria Decision-Making Methods and Artificial Neural Networks

**DOI:** 10.1155/2020/8811834

**Published:** 2020-09-30

**Authors:** Tina Gegovska, Rasit Koker, Tarik Cakar

**Affiliations:** ^1^International Balkan University, Engineering Faculty, Industrial Engineering Dept., Campus Str. Makedonsko Kosovska Brigada bb, Skopje 1000, North Macedonia; ^2^Sakarya University of Applied Sciences, Faculty of Technology, Electrical and Electronical Engineering Dept., Esentepe Campus, Sakarya 54187, Turkey; ^3^Gelisim University, Engineering Faculty Industrial Engineering Dept, Cihangir Mah. Sehit J. Er Hakan Oner Sok. No. 1, Avcilar, Istanbul, Turkey

## Abstract

In recent years, environmental awareness has increased considerably, and in order to decrease endangerments such as air and water pollution, and also global warming, green procurement should be employed. Therefore, in the assessment of suppliers, their environmental performance should be taken into consideration along with other criteria for supplier selection. Raising awareness of sustainability in production and conservation and protection of the environment is very important both for the whole environment and for the company itself by increasing its competitive advantage. And, one of the steps to achieve this is for the companies to try to select green suppliers. So, the purpose of this study is to raise awareness and tackle the need for green supplier selection and, using multiple-criteria decision-making models, to elaborate a case study regarding this. A survey was conducted in a manufacturing firm. The data were analysed, and fuzzy MCDM (multicriteria decision-making) methods and artificial neural networks were implemented. Fuzzy methods are the fuzzy analytic hierarchy process (fuzzy AHP), fuzzy TOPSIS, and fuzzy ELECTRE. ANN supports the result of fuzzy MCDM models from the profit side. ANN can make the best estimate of the current year based on historical data. Fuzzy MCDM methods will also find good solutions using the available data but will produce different solutions as there are different decision-making methods. It is aimed to produce a synergy from the solutions obtained here and to produce a better solution. Instead of a single method, it would be more accurate to produce a better solution than the solution provided by all of them. The dominant result has been obtained using the committee fuzzy MCDM and ANN to select the best green supplier.

## 1. Introduction

In the contemporary world of competitive markets, it is the supply chains that go into competition for a higher place in the international markets. In order to successfully incorporate all of the activities involved in the supply chain, from ordering and supplying of raw materials to manufacturing products and the distribution and transmission to customers, a good supply chain management is needed. Supply chain management (SCM) is an integrated approach to the supply chain's management, plan, and control [1]. Its major aims are to reduce the risk in the supply chain, as well as the production costs, to maximize revenues, and to ameliorate the business processes, the customer service, the inventory levels, and the cycle times. All in all, it helps to increase customer satisfaction, profits, and competitiveness [2]. It is believed that roughly half of the manufacturer's revenues are spent on purchasing goods and services [3]. Thus, it is no wonder that today's consumers demand cheaper, high-quality products, on-time delivery, and admirable services. That is why companies are always trying to lower the costs of their products and materials, but at the same time, they maintain a high-level and first-rate quality and services. The success of the company depends on interactions with suppliers, and that is why the supplier selection process has become extremely important. Supplier selection (SS) is considered the process of finding the right suppliers who could deliver the “right quality products and/or services at the right price, at the right time, and in the right quantities” [4]. However, supplier selection is not an easy task. It is very complicated due to its complexity and the various features and qualifications by different manufacturers. Moreover, in the past decade, an important issue that has been of great concern to the manufacturers is the environmental one. Sustainable operation of companies is emphasized due to the emerging environmental pollution issues. So, it has been established that these issues have to be addressed in the supply chain management, thus setting up the so-called green supply chain management (GSCM). It involves investigating suppliers according to their environmental performance and choosing the one that meets the criteria.

A valuable tool that can be applied to a complex decision like (green) supplier selection is the multicriteria decision-making or MCDM. There are a lot of MCDM models; however, in a vague multiple-criteria decision-making environment when there are ambiguities and uncertainties in the existing information, fuzzy set theory is often applied. In fuzzy MCDM models, the values to evaluate alternatives, which are given in linguistic terms, are represented by fuzzy numbers [5]. Another method that is used when there is vagueness for personal judgement is the artificial neural network (ANN), usually called the neural network, which is an artificial intelligence model that tries to mimic the way the human brain works. As being designed like the way human reasoning functions, ANNs can manage better with complexity and uncertainty than traditional methods. This model is composed of elements like in the biological nervous systems, and they are operating in parallel. The connections between the elements (called neurons) control the function of the network. Thus, connections' values between elements are adjusted, and then the input leads to a particular target output [6].

There are some strategies that organizations have to follow if they want to maintain a competitive position in the global market. Undoubtedly, suppliers are crucial for achieving this competitiveness, and as a result of this, selecting the right suppliers is a vital factor in these strategies. As it was stated a lot of times, supplier selection is a complicated process, and it has been shown to be a multiple-criteria decision-making problem. Due to ecological concerns, there is a tendency for the firms to cooperate with suppliers which are more environmentally oriented, and this is very beneficial for them. Thus, in this paper, “green” (environmental) supplier selection will be implemented in an apparel company in Tetovo. This company has been collaborating with several suppliers; however, a novel method will be applied which might help this company rate higher than its competitors in that industry.

There are a lot of materials and raw materials that are needed for the production of certain apparel, such as fabrics, sewing threads, yarn, trims, and buttons. The quality of the apparel depends on the quality of the raw materials, and that is why the companies must choose their suppliers wisely. For supplying the fabrics, the company works with several suppliers because there are different types of materials and designs needed for different models of apparel. In this study, five suppliers of the twill fabric will be compared.

The simulation models have been developed for the comfortable use of the main roads or when other roads are suitable, and the situation has been examined structurally. Behavior models have been proposed to model this situation in the urban area and to determine parameters and capture travel behavior. In the literature, various behavioral models have been proposed to evaluate the percentage of model selection in urban areas and to capture travel behavior by estimating some suitable parameters. Postorino and Versaci proposed a neurofuzzy approach, especially they used neural networks in nonlinear interactions, and solved the problem by applying the fuzzy logic to the ANN results[7]. A new solution approach is proposed to characterize the defects on metal plates in terms of depth and shape. This problem was solved by making a classification based on words and the fuzzy entropy [8]. Unlike a time-consuming and costly approach, Cacciola et al. proposed [9] a study that allows us to have an idea of 100% probability for mechanical stresses in metallic materials.

The purpose of this study is to tackle the need for green supplier selection and provide a literature review and elaborate a case study in order to find the best green supplier. A survey was conducted in a manufacturing firm in Skopje. Fuzzy multiple-criteria decision-making models (fuzzy AHP, fuzzy TOPSIS, and fuzzy ELECTRE) were implemented, the artificial neural network was formed, and in the end, the results were compared and the best supplier was selected. In this study, the suppliers at hand are listed from the best downwards using the fuzzy MCDM models. Besides, the answer to the question of what sort of ranking would maximize our profit in the past years was searched, and the ranking that maximized the profit was found. Each line of comparison table of each year is given to the artificial neural network as the input, and the supplier ranking that maximizes the profit is taken as the output. The data of the last five years were used in the artificial neural network. A supplier ranking has been obtained by giving the comparison chart of the current year to this trained ANN. Then, the rankings obtained from fuzzy MCDM models and ANN were compared. In each line, the most repeated supplier was taken as the dominant solution, and a single dominant ranking was obtained from these four results.

The trained artificial neural network has contributed significantly to finding a dominant solution to the fuzzy MCDM models as it has learned from the correct rankings of the past years. Thus, instead of obtaining a result by using one fuzzy MCDM model, the results of different fuzzy MCDM models were examined, and a result was obtained by taking lessons from historical data using ANN. Thus, a synergy was created, and the committee of fuzzy MCDM and the ANN solution system was proposed in order to obtain a better solution.

This paper is organized as follows: Section 2 is the literature review. In Section 3, FAHP, FTOPSIS, and FELECTRE calculation steps and mathematical formulations are explained. In Section 4, the numerical applications of FAHP, FTOPSIS, FELECTRE, and artificial neural network have been made for the selection of green terrorists, and the results of each have been obtained. In Section 5, a final solution was found using the solutions obtained for the proposed solution methodology. Conclusion is given in Section 6.

## 2. Literature Review

Supply chain management (SCM) has been a popular topic both in the academic and the industrial circles for many years. It is certain that to have a healthy and long-lasting buyer-supplier relationship, an effective supply chain management is necessary for a company because that will influence its general performance. Purchasing managers should go through periodic supplier performance evaluation, so that they make the right decisions about the continuation of that relationship [10]. Ganeshan and Harrison [11] in their book try to give a definition of what a supply chain is. According to them, it is a system of facilities and alternatives of distributions that implements tasks like obtaining materials, their transformation into finished goods, and their distribution to customers. It is concerned with lowering the production costs, lowering the supply chain's risks, maximizing revenue, improving customer services, and optimizing inventory levels and business processes; which, in turn, brings improved profitability, satisfaction of customers, and competitiveness [2].

Companies, no matter their size, either big or small, have become considerably worried about supply chain management in their endeavour for better quality and higher customer satisfaction. It is now safe to state that companies are no longer in competition between themselves, but between their supply chains. The members should actively and closely collaborate in order to gain competitive advantage. Close relationship and sharing of information is a must between buying firms and their main suppliers. Industrially speaking, SCM involves a variety of managerial and technical matters: the product and process design, efficiently coordinated production of goods, and also contracting suppliers and finding outsourcing, logistics, organization of inventories in various locations, etc. [12]. It is certain that the companies do not depend only on their own performance because the performances of all the units in supply chains affect the company's success. A crucial significance in supply chains is the selection and evaluation of the suppliers, which helps in developing long-term relationships with suppliers, and these relationships are not only dependent on the price of the supplied product, but they influence the company's competitiveness power in a positive way.

### 2.1. Sustainability and Green Supply Chain Management

There are environmental issues like air and water pollution and global warming, and there is no doubt that something should be done about that. The industries themselves are trying to include sustainability practice due to the increased pressure from regulations of the government and also from nongovernmental organizations and the population who are worried about the environment and want to protect it. Thus, companies are trying to balance the economic and environmental performances and gain sustainability. Many researchers have tried to define what sustainability in supply chain management is. Carter and Rogers [13] consider that it is the strategic incorporation and attainment of environmental, social, and economic goals of one organization in the systemic coordination of important interorganizational business issues, with the purpose of ameliorating the long-term economic performance of the particular company, and also its supply chains. Seuring and Muller [14] similarly define sustainable supply chain management and add that in sustainable supply chains, the members of the supply chain themselves should be responsible for fulfilling the environmental and social criteria and competitiveness should be maintained through meeting the appropriate economic criteria and the needs of customers. Environmental pollution issues are more and more emerging, and they are seriously affecting industrial development, so that is why it is crucial that they are addressed in the supply chain management. The supply chain management that deals with environmental issues is called green supply chain management, where the word “green” stands for environment [15]. In general, green supply chain management involves monitoring suppliers according to their environmental performance and then collaborating with the ones that meet appropriate ecofriendly principles and standards [16].

It should be noted that the main goals of the green supply chain management are to decrease the adverse environmental influence during the purchasing of raw materials, production, distribution, and product sales, and in the same time taking care of the waste, as well as products, that are worn out and whose end is near [17]. This is crucial because if there are hazardous substances in the raw materials provided by the suppliers, the whole supply chain will be affected. In addition, as part of the green supply chain management, companies have started developing green products in order to satisfy customer environmental needs and for the purpose of gaining and retaining competitive advantage in the global market [18].

### 2.2. Green Supplier Chain Management

**Figure 1:**
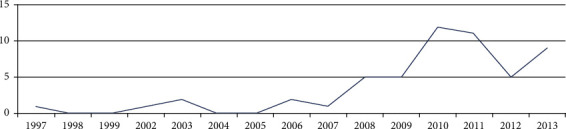
Trend of green supplier selection [19].

Green supplier selection is a key element of a green supply chain management. It is an important activity as the supplier has a significant effect on the supply chain's environmental performance. And, this is important in the sense that many customers would certainly put in a more favorable position the companies that have green consideration. The literature on the traditional supplier selection was elaborated, and since this topic is about the green supplier selection, this area will be in the focus. With the global climate change, academicians and managers started focusing on the environmental feature, and Figure 1 shows the growth of green supplier selection from the appearance to 2013.

The criteria that have been considered by some of the researchers are given in the following. Lee et al. [20] in their research used fuzzy AHP and took into consideration quality, technology capability, total product life cycle cost, and also the green criteria like green image, pollution control, environment management, green product, and green competencies. Grisi et al. [21] considered the following criteria as suitable for assessment in green supplier selection: availability of “clean” technologies, ecological materials, environmental policies, environmental planning, ISO 14001, green image, and current environmental impact. For Chen et al. [22], the criteria to be considered for green supplier selection were quality, delivery, flexibility, green design, green purchasing, life cycle assessment, ISO 14000 certificates, R&D green products, cleaner production, and environmental management system. Kuo and Lin [23] for the supplier selection took into consideration environmental administration system, environmental system, and environmental planning and green purchasing and implemented it with analytic network process (ANP) and data envelopment analysis (DEA). Kannan et al. [24] in their research included environment protection, corporate social responsibility, pollution control, green product, green image, green innovation, and hazardous substance management as criteria in the green supplier selection. Rouyendegh et al. [25] addressed the problem of GSS, aiming to obtain environmental sensitivity, sustainability, and durability.

Some researchers, like those mentioned earlier, think that in selecting a supplier both environmental and economic scopes should be simultaneously considered [26]. However, in the literature, there are researchers that consider only the environmental dimensions. Govindan et al. [27] have noted that there is an abundance of literature on supplier selection, and only a slight amount on green supplier selection and definition on green criteria. Also, in their research where they reviewed 33 papers, they have found that both traditional and environmental criteria have been used to evaluate and select suppliers, and in Table 1, the top ten criteria used for green supplier selection can be seen.

Also, Nielsen et al. [19] in their research analysed environmental criteria similarly as by Hsu and Hu [28], and the results of the frequencies in both research papers are given in Table 2.

### 2.3. Supplier Selection Methods

In the literature, numerous supplier selection methods are available. Zhang et al. [29] separate supplier selection methods into 5 categories: linear weighting models, mathematical programming models, statistical approach, artificial intelligence-based models, and cost-based models. Kannan et al. [30] studied the literature on application of MCDM techniques for supplier selection published from 2008 to 2012 and found that the most popular were analytic hierarchy process (AHP), analytic network process (ANP), linear programming (LP), and data envelopment analysis (DEA). Similarly, there is an abundance of decision-making tools used in the literature about green supplier selection problems. Like Chai et al. [31], Govindan et al. [28] studied the tools used for green supplier selection in their (previously mentioned) review of 33 papers where they also studied the criteria used. They found that for green supplier selection, the most popular approach was analytic hierarchy process (27.78%) and then analytic network process (16.6%), data envelopment analysis (11.1%), linear programming (8.76%), TOPSIS (5.56%), and multiobjective optimization (2.77%).

Some examples of the research studies and the tools they used are as follows. AHP is the most used approach, and one research where this approach is applied is the one by Handfield et al. [32] where they assess the relative significance of some environmental characteristics and see the relative performance of the suppliers along these characteristics. Hsu and Hu [28, 33] in two of their works used ANP and applied it in the electronic industry. Wu and Blackhurst [34] evaluated suppliers and their green performance by using the DEA approach. Kannan et al. [30] used the fuzzy TOPSIS approach for green supplier evaluation and applied it to an electronics company in Brazil. Uygun and Dede [35] developed an MCDM model using the fuzzy decision-making trial and evaluation laboratory (DEMATEL), fuzzy analytical network process (ANP), and fuzzy TOPSIS methods. Wang et al. [36] evaluated both economic and environmental criteria for green supplier selection by using fuzzy AHP together with fuzzy TOPSIS. Banaeian et al. [37] for green supplier selection in the agrifood industry applied fuzzy MCDM approaches: fuzzy TOPSIS, fuzzy VIKOR, and fuzzy grey relational analysis (GRA). Sustainable supplier selection is strategically important and is a critical stage for the sustainable supply chain. The working stages of the supply chain directly depend on this activity. Durmić [38] identified the most important criteria for the selection of a sustainable supplier in the company for lime production. In this process, a team of experts has been created to compare criteria grouped at two levels for decision-making. The full consistency method (FUCOM) was applied to determine the importance of the criteria. Đalić et al. [39] proposed the fuzzy rough MCDM model. Model's name was Fuzzy PIvot Pairwise RElative Criteria Importance Assessment—Fuzzy PIPRECIA. Nine environmental criteria-based evaluations were made, and an example of the model representing supplier selection was proposed. The fuzzy PIPRECIA method is used to determine the importance of the given seven criteria: CR1—environmental image, CR2—recycling, CR3—pollution control, CR4—environmental management system, CR5—environmentally friendly products, CR6—resource consumption, and CR7—green competencies. Petrović et al. [40] used three different fuzzy multicriteria decision-making (MCDM) methods for supplier selection. The methods used are fuzzy TOPSIS, fuzzy WASPAS, and fuzzy ARAS methods. A new solution was obtained by looking at the results of these three methods. Chatterjee and Stević [41] proposed a two-step solution for supplier selection. In the first stage, they listed the suppliers using FAHP. Then, by taking a certain number from the solution ranking given by FAHP, they applied FTOPSIS to them and ranked the suppliers according to their performance. There was only one research found that applied the artificial neural network for evaluating green suppliers and that was the research by Chen et al. [22] where the artificial neural network is combined with MADA methods for helping in a green supplier selection. In this study, for the process of green supplier selection, some fuzzy multicriteria decision-making methods: fuzzy AHP, fuzzy TOPSIS, and fuzzy ELECTRE, and also artificial neural network, will be applied in order to compare the findings.

## 3. Formulations of Fuzzy MCDM

### 3.1. Fuzzy Analytic Hierarchy Process (Fuzzy AHP)

In order to introduce the fuzzy analytic hierarchy process, analytic hierarchy process has to be known. AHP was developed by Saaty [42], an American mathematician working at the University of Pittsburgh.

The AHP is a widely used method in multicriteria decision-making problems. As defined by DSS Resources (n.d.), it is an approach for making decisions in which multiple criteria are structured into a hierarchy and their relative importance is assessed. The analyst makes pairwise comparisons of alternatives for each criterion and determines the alternatives' ranking. However, this method is insufficient to explain the impreciseness of human's judgement because sometimes the nature of the criteria is subjective or qualitative, and their opinions cannot be represented as exact numbers [43]. So, the uncertainty and vagueness in the decision makers' opinions can be controlled through fuzzy set theory, with fuzzy AHP. This method is an improvement of a standard AHP method using the fuzzy logic approach that in the calculations uses fuzzy numbers instead of real ones [44]. Fuzzy analytic hierarchy process was proposed by Chang [45]. Fuzzy AHP has been used in various fields, like selection of personnel, energy alternatives, jobs, and even weapon selection, but it has been mostly used for supplier selection. Among the first researchers who used fuzzy AHP were van Laarhoven and Pedrycz [46] who extended Saaty's model into the fuzzy domain, and they defined the triangular membership functions for the pairwise comparisons. Later on, it was Buckley [47] who determined the fuzzy priorities of comparison ratios in the triangular membership function, and his methods will be used in this paper. Cakar and Shabani [48] used fuzzy AHP, fuzzy TOPSIS, and fuzzy ELECTRE for personal selection. There are several steps that the analyst has to take into consideration for a solution of a decision problem with FAHP [49].

**Step 1 .**

Comparison of criteria or alternatives.

The decision makers make comparison of criteria and alternatives through linguistic terms, and these terms match the separate fuzzy triangular numbers. They are given in Table 3.

Comparing criterion *n* to criterion *m* ⟶ (l, *m*, u).

Comparing criterion *m* to criterion *n* ⟶ (1/*u*, 1/*m*, 1/*l*).

So, if the decision maker thinks that criterion 1 is fairly more important than criterion 2, the fuzzy triangular number (4, 5, 6) will be used, and then, when comparing the other way around, criterion 2 to criterion 1, the fuzzy triangular number (1/6, 1/5, 1/4) will be used.

**Step 2 .**

A pairwise contribution matrix is formed.

The model of a pairwise contribution comparison matrix is shown below:

(1) $$\begin{array}{c} \left[\begin{array}{cccc} {\tilde{d}}_{11}^{k} & {\tilde{d}}_{12}^{k} & \ldots & {\tilde{d}}_{1n}^{k}\\ {\tilde{d}}_{21}^{k} & {\tilde{d}}_{21}^{k} & \ldots & {\tilde{d}}_{2n}^{k}\\ . & . & . & .\\ . & . & . & .\\ . & . & . & .\\ {\tilde{d}}_{n1}^{k} & {\tilde{d}}_{n2}^{k} & \ldots & {\tilde{d}}_{nn}^{k} \end{array}\right], \end{array}$$

where ${\tilde{d}}_{ij}^{k}$ indicates the *k* ^th^ decision maker's preference of the *i* ^th^ criterion over the *j* ^th^ criterion, and they are fuzzy triangular numbers which is indicated by the tilde “∼” above the symbol.

**Step 3 .**

Averaging the preferences if there are more decision makers.

If there is more than one decision maker, the preferences are averaged, and it is indicated with the symbol $\tilde{d_{ij}}$ and calculated as

(2) $$\begin{array}{c} \overset{˜}{d_{ij}}=\;\frac{\sum_{k=1}^{K}\overset{˜}{d_{ij}^{k}}}{K}. \end{array}$$

**Step 4 .**

Updating the pairwise contribution matrix.

The pairwise contribution matrix is updated according to the averaged decision makers' preferences, and it looks like the following:

(3) $$\begin{array}{c} \tilde{A}=\left[\begin{array}{ccc} \tilde{d_{11}} & \cdots & \tilde{d_{1n}}\\ \vdots & \ddots & \vdots \\ \tilde{d_{n1}} & \cdots & \tilde{d_{nn}} \end{array}\right]. \end{array}$$

**Step 5 .**

Calculating the geometric mean of the fuzzy values of each criterion.

For each criterion, the geometric mean of the fuzzy comparison values is calculated according to

(4) $$\begin{array}{c} \tilde{r_{i}}={\left(\overset{n}{\underset{j=1}{\prod }}\tilde{d_{ij}}\right)}^{1/n},\;i=1,2,\ldots ,n. \end{array}$$

**Step 6 .**

Fuzzy weights ($\tilde{w_{i}}$) for each criterion are calculated.

(5) $$\begin{array}{c} \tilde{w_{i}}=\tilde{r_{i}}\times {\left(\tilde{r_{1}}|+|\tilde{r_{2}}|+|\ldots |\tilde{r_{n}}\right)}^{-1}=\left(lw_{i},mw_{i},uw_{i}\right), \end{array}$$

- First, the vector summation of each $\tilde{r_{i}}$ should be found
- The reverse value (power of −1) of the summation vector is calculated
- The fuzzy triangular number that is obtained is arranged in an increasing order
- Each $\tilde{r_{i}}$ is multiplied with this reverse vector, and the fuzzy weight of criterion *i* is obtained

**Step 7 .**

Defuzzifying the fuzzy triangular numbers of the fuzzy weight.

This is done by the centre of the area method which was proposed by Chou and Chang [50] in order to get a single number, by applying the following equation:

(6) $$\begin{array}{c} M_{i}=\frac{lw_{i}+\;mw_{i}+\;uw_{i}}{3}. \end{array}$$

**Step 8 .**

Normalizing the nonfuzzy number *M*_*i*_.

The nonfuzzy number *M*_*i*_ needs to be normalized, and that is done by the following equation:

(7) $$\begin{array}{c} N_{i}=\;\frac{M_{i}}{\sum_{i=1}^{n}M_{i}}. \end{array}$$

The normalized weights of the criteria and the alternatives are calculated in this way.

**Step 9 .**

Each alternative weight is multiplied with the related criteria to calculate the scores of the alternatives.

When alternative weights are multiplied with the related criteria, the scores for each alternative are obtained, and the alternative that has the highest score is chosen.

### 3.2. Fuzzy TOPSIS

TOPSIS (technique for order preference by similarity to ideal solution) was proposed in 1980 by Hwang and Yoon [51]. TOPSIS is a multiple-criteria decision-making (MCDM) method which is used for identification of a solution from a limited set of alternatives. The idea of the TOPSIS method is to identify the shortest distance to the ideal solution and the furthest distance from the anti-ideal solution [52]. This ideal solution, also called as a positive ideal solution, maximizes the benefit metrics (criteria/attributes) and minimizes the cost metrics. On the other hand, the negative ideal solution, also called as the anti-ideal solution, maximizes the cost metrics and minimizes the benefit metrics [53].

However, usually, the assigned decision maker's performance ratings are imprecise, and that is why the fuzzy TOPSIS is preferred, the one that uses fuzzy triangular numbers. Thus, the optimal solution will be the one that is nearest to the fuzzy positive ideal solution (FPIS) and furthest from the fuzzy negative ideal solution (FNIS). How fuzzy TOPSIS works is given according to that by Sodhi and Prabhakar [54]:

*Step 1*. The *k* ^th^ decision maker gives fuzzy rating and importance weight about the *i* ^th^ alternative on the *j* ^th^ criterion, for the fuzzy number (*a*, *b*, *c*):
  (8) $$\begin{array}{c} \tilde{x_{ij}^{k}}=\left(a_{ij}^{k},b_{ij}^{k},c_{ij}^{k}\right),\\ \tilde{w_{ij}^{k}}=\left(w_{j1}^{k},w_{j2}^{k},w_{j3}^{k}\right),\;\mathrm{where}\;i=1,2,\ldots ,m\;\mathrm{and}\;j=1,2,\ldots n. \end{array}$$

*Step 2*. Obtaining the aggregated fuzzy rating and aggregated fuzzy weight.
The aggregate fuzzy ratings $\tilde{x_{ij}}$, where $\tilde{x_{ij}}=\left(a_{ij},b_{ij},c_{ij}\right)$, will be obtained in the following manner:
  (9) $$\begin{array}{c} a_{ij}=\min_{k}\left\{a_{ij}^{k}\right\}\;,\\ b_{ij}=\frac{1}{K}\overset{K}{\underset{k=1}{\sum }}b_{ij}^{k},\\ c_{ij}=\min_{k}\left\{c_{ij}^{k}\right\}. \end{array}$$

On the other hand, the aggregate fuzzy weights $\tilde{w_{ij}}$ of each criterion, where $\tilde{w_{j}^{k}}=\left(w_{j1},w_{j2},w_{j3}\right)$, are calculated in the following manner:
  (10) $$\begin{array}{c} w_{j1}=\min_{k}\left\{w_{jk1}\right\}, \end{array}$$
  (11) $$\begin{array}{c} w_{j2}=\frac{1}{K}\overset{K}{\underset{k=1}{\sum }}w_{jk2}, \end{array}$$
  (12) $$\begin{array}{c} w_{j3}=\max_{k}\left\{w_{jk3}\right\}. \end{array}$$

*Step 3*. The fuzzy decision matrix is constructed.
A fuzzy multicriteria group decision-making problem can be illustrated as follows: 
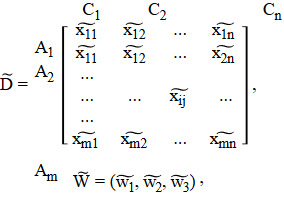
     (13)

where $\tilde{x_{ij}}\;\forall_{i,\;j}$ and $\tilde{w_{j}}=1,2,\ldots ,m$ and *j*=1,2,…, *n* are linguistic variables that can be described by fuzzy triangular numbers, $\tilde{x_{ij}}=\left(a_{ij},b_{ij},c_{ij}\right)$ and $\tilde{w_{j}}=\left(w_{j1},w_{j2},w_{j3}\right)$.
*Step 4*. A normalized fuzzy decision matrix is formed:
  (14) $$\begin{array}{c} \tilde{R}=\left[\tilde{r_{ij}}\right]\text{mxn},\;i=1,2,\ldots ,m,\;\mathrm{and}\;j=1,2,\ldots ,n\;\text{where,}\\ \tilde{r_{ij}}=\left(\frac{a_{ij}}{c_{j}^{*}},\;\frac{b_{ij}}{c_{j}^{*}}\frac{c_{ij}}{c_{j}^{*}},\right)\;\mathrm{and}\;c_{j}^{*}=\max\left\{a_{ij}\right\}\;\mathrm{for}\text{ the benefit criteria,}\\ \tilde{r_{ij}}=\left(\frac{a_{j}^{-}}{c_{ij}}a_{j}^{-}/a_{j}^{-}/b_{ij},a_{j}^{-}/a_{ij}\right)\;\mathrm{and}\;a_{j}^{-}=\max_{i}\left\{a_{ij}\right\}\;\mathrm{for}\text{ the cost criteria}. \end{array}$$

*Step 5*. Constructing the weighted normalized fuzzy decision matrix $\tilde{V}$.
Multiplying the evaluation criteria's weights ($\tilde{w_{j}}$) with the normalized fuzzy decision matrix ($\tilde{r_{ij}}$), the weighted normalized fuzzy decision matrix is obtained:
  (15) $$\begin{array}{c} \tilde{V}=\left[v_{ij}\right]m\times n,\;i=1,2,\ldots ,m\;\mathrm{and}\;j=1,2,\ldots ,n,\;\mathrm{where}\\ v_{ij}=r_{ij}\times w_{j}. \end{array}$$

*Step 6*. Determining FPIS and FNIS and calculating the distance of each alternative from FPIS and FNIS, respectively.
FPIS (*A* ^*∗*^) and FNIS (*A* ^*−*^) of the alternatives are calculated as follows:
  (16) $$\begin{array}{c} A^{*}=\left(\tilde{v_{1}^{*}},\tilde{v_{2}^{*}},\tilde{v_{3}^{*}}\right)\;\mathrm{where}\;v_{j}^{*}=\max\left(v_{ij3}\right),\;i=1,2,\ldots ,m\;\mathrm{and}\;j=1,2,\ldots ,n, \end{array}$$
  (17) $$\begin{array}{c} A^{-}=\left(\tilde{v_{1}^{-}},\tilde{v_{2}^{-}},\tilde{v_{3}^{-}}\right)\;\mathrm{where}\;v_{j}^{-}=\min\left(v_{ij1}\right),\;i=1,2,\ldots ,m\;\mathrm{and}\;j=1,2,\ldots ,n. \end{array}$$

Moreover, the distance (*d*_*i*_^*∗*^ and *d*_*i*_^−^) of each weighted alternative *i* = 1, 2,…, *m* from the FPIS and the FNIS is obtained as follows:
  (18) $$\begin{array}{c} d_{i\;}^{*}=\overset{n}{\underset{j=1}{\sum }}d_{v}\;\left(\tilde{v_{ij}},\;\tilde{v_{j}^{*}}\right),\;i=1,2,\ldots ,m, \end{array}$$
  (19) $$\begin{array}{c} d_{i}^{-}=\overset{n}{\underset{j=1}{\sum }}d_{v}\;\left(\tilde{v_{ij}},\;\tilde{v_{j}^{-}}\right),\;i\;=\;1,2,\ldots ,m, \end{array}$$

  where $d_{v}\left(\tilde{a},\tilde{b}\right)$ is the distance measurement between the two fuzzy numbers $\tilde{a}$ and $\tilde{b}$.
 
*Step 7*. Calculation of the closeness coefficient of each alternative is done, and then the alternatives are ranked according to this coefficient.

The closeness coefficient *CC*_*i*_ represents the distances to the fuzzy positive ideal solution, *A* ^*∗*^, and the fuzzy negative ideal solution, *A* ^*−*^, simultaneously. It is calculated according to the following equation:

(20) $$\begin{array}{c} CC_{i}=\frac{d_{i}^{-}}{d_{i}^{-}+\;d_{i\;}^{*}},\;i=1,2,\ldots ,m. \end{array}$$

As for the ranking, the alternative with the highest closeness coefficient represents the best alternative and is closest to the FPIS and farthest from the FNIS.

### 3.3. Fuzzy ELECTRE

The ELECTRE (elimination and choice translating reality English, English translation from the French original, **EL**imination **E**t **C**hoix **T**raduisant la **RE**alité) method was first introduced in 1966 by Benayoun [55]. The ELECTRE method is concerned with “outranking relations” by using alternatives' comparisons in pairs according to each criterion [56]. Alternatives are considered as dominated, if another alternative surpasses them in one or more attributes and equals in the other attributes. This method is founded based on a concordance and a discordance set, which can be viewed as measurements of dissatisfaction that a decision maker uses to choose an alternative. With the ELECTRE method, less favorable alternatives are eliminated, which is very suitable in decision-making when encountering a few criteria with a large number of alternatives, thus giving a clearer view of alternatives [57]. Usually, the decision makers evaluate the criteria and alternatives in linguistic and qualitative values, and so the fuzzy ELECTRE comes forward, which uses the fuzzy triangular numbers. The following steps best illustrate the fuzzy ELECTRE, according to Ali [58]:

*Step 1*. Fuzzy decision matrix ($\tilde{X}$) is constituted: 
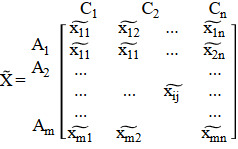
     (21)

where *C*_1_, *C*_2_,…, *C*_*n*_ are the decision-making criteria, *A*_1_, *A*_2_,…, *A*_*n*_ are the alternatives, and $\tilde{x_{ij}\;}=\left(\tilde{x_{ij}^{a}},\tilde{x_{ij}^{b}},\tilde{x_{ij}^{c}}\right)$ is the fuzzy value of *A*_*i*_ in the criterion *C*_*j*_.
*Step 2*. The normal decision matrix ($\tilde{N}$) is constituted.
Each of the elements of the normal decision matrix is obtained according to the following equation:
  (22) $$\begin{array}{c} \overset{˜}{n_{ij}}=\frac{\overset{˜}{x_{ij}}}{\sqrt{\sum_{i=1}^{m}{\left(s\left(\tilde{x_{ij}},\;0\right)\right)}^{2}}},\;j=1,2,\ldots ,n, \end{array}$$

where $\tilde{n_{ij}}=\left(\tilde{n_{ij}^{a}},\tilde{n_{ij}^{b}},\tilde{n_{ij}^{c}}\right)$ is the normalized value of $\tilde{x_{ij}\;}=\left(\tilde{x_{ij}^{a}},\tilde{x_{ij}^{b}},\tilde{x_{ij}^{c}}\right)$ and
  (23) $$\begin{array}{c} s\left(\tilde{x_{ij}},\;0\right)=\frac{x_{ij}^{a}+\;2x_{ij}^{b}+\;x_{ij}^{c}}{4}. \end{array}$$

It should be noted that equation (19) is derived from the equation about the distance between two fuzzy numbers:
  (24) $$\begin{array}{c} s\left(\tilde{x},\tilde{y}\right)=\frac{\left(x_{1}+2x_{2}+\;x_{3}\right)-\;\left(y_{1}+\;2y_{2}+\;y_{3}\right)}{4}. \end{array}$$

*Step 3*. Weighted matrix ($\tilde{V}$) is constituted.
Depending on the values from the matrix for the weights of criteria, there are two ways to obtain the values of the weighted matrix $\tilde{v_{ij}}=\left(v_{ij}^{a},v_{ij}^{b},v_{ij}^{c}\right)$:
  (1) If the values of the matrix for the weights of criteria are single numbers *W*=[*w*_1_, *w*_2_,…, *w*_*n*_], the values of the weighted matrix will be obtained according to
    (25) $$\begin{array}{c} \tilde{v_{ij}}=\left(w_{j}\times n_{ij}^{a},w_{j}\times n_{ij}^{b},w_{j}\times n_{ij}^{c}\right), \end{array}$$
  where *i*=1,2,…, *m*, *j*=1,2,…, *n* and *w*_*j*_ is the weight of *j*th criterion and ∑_*j*−1_^*n*^ *w*_*j*_=1.
  (2) If the values of the matrix for the weights of criteria are triangular fuzzy numbers [$w=\tilde{w_{1}},\tilde{w_{2}},\ldots ,\tilde{w_{n}}$], the values of the weighted matrix will be obtained according to
    (26) $$\begin{array}{c} \tilde{v_{ij}}=\left(s\left(\tilde{w_{j}},0\right)x\right)n_{ij}=\left(\left(s\left(\tilde{w_{j}},0\right)x\right)n_{ij}^{a},\left(s\left(\tilde{w_{j}},0\right)x\right)n_{ij}^{b},\left(s\left(\tilde{w_{j}},0\right)x\right)n_{ij}^{c}\right), \end{array}$$

where *i*=1,2,…, *m*, *j*=1,2,…, *n*, and *w*_*j*_ is the weight of the *j* th criterion and $\sum_{j-1}^{n}s\left(\tilde{w_{j}},0\right)=1;s\left(\tilde{w_{j}},0\right)\geq 0,\;j=1,2,\ldots ,n$.
*Step 4*. Determination of concordance (*S*_*kl*_) and discordance (*D*_*kl*_) sets.
The set of the existing *j* criteria is divided into two subsets: concordance (*S*_*kl*_) and discordance (*D*_*kl*_).
The concordance set *S*_*kl*_ from two choices *A*_*k*_ and *A*_*L*_ will contain the criteria for which *A*_*k*_ is privileged over *A*_*L*_. Actually,
  (27) $$\begin{array}{c} S_{KL}=\;\left\{j\;\begin{array}{c} s\;\left(\tilde{v_{Kj},}\;0\right)\geq \;s\;\left(\tilde{v_{Lj},}\;0\right)\\ s\;\left(\tilde{v_{Kj},}\;0\right)\leq \;s\;\left(\tilde{v_{Lj},}\;0\right) \end{array}\right\}. \end{array}$$

The discordance set *D*_*kl*_ from two choices *A*_*k*_ and *A*_*L*_ will contain the criteria for which
  (28) $$\begin{array}{c} D_{KL}=\;\left\{j\;\begin{array}{c} s\;\left(\tilde{v_{Kj},}\;0\right)<\;s\;\left(\tilde{r_{Lj},}\;0\right)\\ s\;\left(\tilde{v_{Kj},}\;0\right)<\;s\;\left(\tilde{r_{Lj},}\;0\right) \end{array}\right\}=J-S_{KL}. \end{array}$$

*Step 5*. Fuzzy concordance matrix ($\tilde{I_{\text{KL}}}$) is constituted.
For constituting the fuzzy concordance matrix, concordance sets are used. The fuzzy concordance criterion will be equal to the sum of $\tilde{w_{j}}$ weights of criteria that are part of the concordance *S*_*KL*_ set. The elements of the matrix are calculated by the help of the relation which is shown in the equation below:
  (29) $$\begin{array}{c} \tilde{I_{\text{KL}}}=\underset{j\in S_{\text{KL}}}{\sum }\tilde{w_{j}}, \end{array}$$

where
  (30) $$\begin{array}{c} \overset{n}{\underset{j-1}{\sum }}S\left(\tilde{w_{j}},0\right)=1. \end{array}$$

The consecutive values from the $\tilde{I_{\text{KL}}}$ criteria (*K, L* = 1, 2,…, *n*; *L* ≠ *K*) form an asymmetrical fuzzy concordance matrix $\tilde{I}$ which looks like the following:
  (31) $$\begin{array}{c} \tilde{I}=\left[\begin{array}{cccccc} - & \tilde{I_{12}} & \tilde{I_{13}} & \ldots & \tilde{I_{1\left(n-1\right)}} & \tilde{I_{1n}}\\ \tilde{I_{21}} & - & \tilde{I_{23}} & \ldots & \tilde{I_{2\left(n-1\right)}} & \tilde{I_{2n}}\\ . & . & . & . & . & .\\ . & . & . & . & . & .\\ . & . & . & . & . & .\\ \tilde{I_{m1}} & \tilde{I_{m2}} & \tilde{I_{m3}} & \ldots & \tilde{I_{m\left(n-1\right)}} & - \end{array}\right]. \end{array}$$

*Step 6*. Fuzzy discordance matrix ($\tilde{{\text{NI}}_{KL}}$) is constituted.
For constituting the fuzzy discordance matrix, discordance sets are used, and also the elements of the weighted fuzzy decision matrix $\tilde{V}$. The elements of this matrix are calculated as follows:
  (32) $$\begin{array}{c} \tilde{{\text{NI}}_{KL}}=\;\frac{\text{GH}\left(\tilde{V_{Kt^{\prime }}},\tilde{V_{Lt\prime }}\right)}{S\left(\tilde{V_{Kt}}\;,\tilde{V_{Lt}}\right)}, \end{array}$$

where
  (33) $$\begin{array}{c} \text{GH}\left(\tilde{V_{Kt^{\prime }}}\;,\tilde{V_{Lt^{\prime }}}\right)=\left\{\begin{array}{cc} \tilde{V_{Kt^{\prime }}}-\tilde{V_{Lt^{\prime }}}, & \mathrm{if}\;S\left(\tilde{V_{Kt^{\prime }}},\tilde{V_{Lt^{\prime }}}\right)\geq 0,\\ \tilde{V_{Lt^{\prime }}}-\tilde{V_{Kt^{\prime }}}, & \text{else,} \end{array}\right. \end{array}$$

*t′* is a value of the *D*_*KL*_ set, for which $\left|S\left(\tilde{V_{Kt^{\prime }}},\tilde{V_{Lt^{\prime }}}\right)\right|$ is at its maximum value; *t* is a value of the {1, 2,…, *n*} set, for which $\left|S\left(\tilde{V_{Kt}},\tilde{V_{Lt}}\right)\right|$ is at its maximum value.
And, we get an asymmetrical fuzzy discordance matrix $\tilde{\text{NI}}$ which looks like the following:
  (34) $$\begin{array}{c} \tilde{\text{NI}}=\;\left[\begin{array}{cccccc} - & \tilde{{\text{NI}}_{12}} & \tilde{{\text{NI}}_{13}} & \ldots & \tilde{{\text{NI}}_{1\left(n-1\right)}} & \tilde{{\text{NI}}_{1n}}\\ \tilde{{\text{NI}}_{21}} & - & \tilde{{\text{NI}}_{23}} & \ldots & \tilde{{\text{NI}}_{2\left(n-1\right)}} & \tilde{{\text{NI}}_{2n}}\\ . & . & . & . & . & .\\ . & . & . & . & . & .\\ . & . & . & . & . & .\\ \tilde{{\text{NI}}_{m1}} & \tilde{{\text{NI}}_{m2}} & \tilde{{\text{NI}}_{m3}} & \ldots & \tilde{{\text{NI}}_{m\left(n-1\right)}} & - \end{array}\right]. \end{array}$$

 
*Step 7*. The effective concordance matrix (*F*) is constructed.

The values from the concordance matrix $\tilde{I_{KL}}$ should be measured against a common threshold value $\tilde{\text{Ī}}$ to observe the privilege that *A*_*K*_ has over *A*_*L*_. The privilege is bigger when $\tilde{I_{KL}}$ exceeds the minimum threshold $\tilde{\text{Ī}}$:

(35) $$\begin{array}{c} \tilde{I_{KL}}\geq \tilde{\text{Ī}},\\ S\left(\tilde{I_{KL}},\tilde{\text{Ī}}\right)\geq 0. \end{array}$$

$\tilde{\text{Ī}}$ is a desired value but usually is presented as the mean value of the concordance criteria:

(36) $$\begin{array}{c} \tilde{\text{Ī}}=\overset{m}{\underset{K=1}{\sum }}\overset{m}{\underset{L=1}{\sum }}\frac{\tilde{I_{KL}}}{m\left(m-1\right)}. \end{array}$$

The matrix is constructed based on the minimum threshold, and it contains the elements *f*_*ij*_ that are either 0 or 1 (1 meaning priority of one choice over another), depending on the following:

(37) $$\begin{array}{c} \left\{\begin{array}{cc} f_{KL}=1,\; & \mathrm{if}\;S\left(\tilde{I_{KL}},\tilde{\text{Ī}}\right)\geq 0,\\ f_{KL}=0, & \mathrm{else}\text{.} \end{array}\right. \end{array}$$

  Step *8*. The effective discordance matrix (*G*) is constructed.

The values from the discordance matrix $\tilde{{\text{NI}}_{KL}}$ should be measured against a threshold value $\tilde{\text{NĪ}}$. The threshold value is obtained as

(38) $$\begin{array}{c} \tilde{\text{NĪ}}=\overset{m}{\underset{K=1}{\sum }}\overset{m}{\underset{L=1}{\sum }}\frac{\tilde{{\text{NI}}_{KL}}}{m\left(m-1\right)}. \end{array}$$

The matrix is constructed based on the minimum threshold, and it contains the elements *g*_*ij*_ that are either 0 or 1 (1 meaning priority of one choice over another), depending on the following:

(39) $$\begin{array}{c} \left\{\begin{array}{cc} g_{KL}=1,\; & \mathrm{if}\;S\left(\tilde{{\text{NI}}_{KL}},\;\tilde{\text{NĪ}}\right)\leq 0,\\ g_{KL}=0, & \text{else}. \end{array}\right. \end{array}$$

 
*Step 9*. General matrix (H) is constituted.

The elements *h*_*ij*_ of the general matrix H are equal to the reciprocal multiplication of *f*_*ij*_ and *g*_*ij*_ elements, and it is constituted of either 1 or 0 value:

(40) $$\begin{array}{c} h_{ij}=f_{ij}\times g_{ij}. \end{array}$$

This matrix shows the relative privileges in choices. Namely, if *h*_*KL*_ = 1, *A*_*K*_ is privileged over *A*_*L*_, both in terms of concordance and discordance criterion, and still be dominated by others.

 
*Step 10.* The less attractive choices are eliminated.

The general matrix gives us a certain look into the choices. The condition for *A*_*K*_ to be an effective choice is the following:

(41) $$\begin{array}{c} \mathrm{for at least one}\;l,\;h_{KL}=1,\;\mathrm{such that}\;l=1,2,\ldots ,m;l\neq k,\\ \mathrm{for every}\;i,\;h_{IK}=0\;\mathrm{such that}\;l=1,2,\ldots ,m;i\neq k,i\neq l. \end{array}$$

## 4. Implementation

In recent years, the need for supply chain management has increased, which is due to customer demands, market competition, advancement of technology, and decreases in governmental regulations. Another thing that influences the need for supply chain management is the rise in the environmental consciousness [59, 60]. Thus, there are a lot of things that have to be considered, and selecting the right supplier is an important issue that the firm's management is faced with. The aim of this study is to find the best supplier of the twill fabric out of the five in question and compare the results obtained from the different methods used. Different criteria, both conventional and environmental, were considered.

### 4.1. Formulation of the Decision Criteria

To select which criteria will be used in this case, a questionnaire was designed and distributed to six experts in the environment and supply chain. The questionnaire, given in Appendix A, is based on the Likert scale representing the importance of each criterion (“5—extremely important,” “4—very important,” “3—moderately important,” “2—slightly important,” and “1—not at all important”). If a criterion has a lower total score, it is omitted. As a result, the supplier evaluation criteria were decided. From the traditional criteria, quality, cost, delivery, and service were taken into consideration. On the other hand, pollution control, green product, and environmental management were used as environmental criteria. The criteria and the subcriteria are given in Table 4.

According to these criteria and their subcriteria, twelve people, both from the management and the employees, evaluated the five suppliers. Moreover, separate comparison will be made for the requirements of the methods which will be used.

### 4.2. Implementation of Fuzzy Analytic Hierarchy Process (Fuzzy AHP)

The fuzzy analytical hierarchy process (FAHP) is one of the most commonly applied methods in practice. Like the regular AHP, it helps the decision makers to arrange the criteria and alternatives into a decision hierarchy, consisting of three levels: criteria, alternatives, and goals. The only difference is that fuzzy numbers are used in order to avoid the ambiguousness of the assessment of the suppliers.

Evaluation of the criteria (Appendix B) and the alternatives according to each criterion (Appendix C) is made by the managers and staff of the firm. According to the evaluations, comparison matrices are formed by using the values from Table 5. Later on, the pairwise contribution matrix of the criteria is formed according to the comparisons between the criteria. Then, the geometric mean of the fuzzy values for each criterion is taken, which helps in the calculation of the weights. The next step includes defuzzifying the fuzzy triangular numbers and normalizing the results from the defuzzification (which are now nonfuzzy numbers). These will be weights of the criteria. Then, comparisons of each supplier are made according to each criterion, and the pairwise contribution matrices are constructed for each alternative (supplier). The same steps are implemented to get the normalized weights for each alternative. Later on, each alternative weight is multiplied with the related criteria in order to calculate the scores of the alternatives. The illustration of the calculations from the firm's data in practice: first, the pairwise contribution matrix of the criteria (Table 5) is given, according to the pairwise comparisons of the criteria, with the inquiry form in Appendix B.

The geometric mean of the fuzzy values of each criterion is calculated by equation (4), and the following results were obtained.

For example, for quality:

(42) $$\begin{array}{c} \tilde{r_{1}}={\left(\overset{7}{\underset{j=1}{\prod }}\tilde{d_{1j}}\right)}^{1/7}=\left[{\left(1*1*4*2*2*4*1\right)}^{1/7};\;{\left(1*1*5*3*3*5*1\right)}^{1/7};{\left(1*1*6*4*4*6*1\right)}^{1/7}\right]=\left(1.811,2.168,2.479\right). \end{array}$$

The geometric means of the fuzzy comparison values of all criteria are shown in Table 6.

Moreover, the total values and the reverse values of these means should be also presented. They are given in Table 7 together with a new order of the fuzzy number since the fuzzy triangular number should be in the increasing order.

In the next step, the relative fuzzy weights *ῶ*_*i*_ are calculated in the following manner, according to equation (5):

(43) $$\begin{array}{c} \omega 1=\left[\left(1,8114*0,0956\right);\left(2,1678*0,1147\right);\left(2,4793*0,1412\right)\right]=\left[\left[0,0522;0,0753;0,1125\right.\right]. \end{array}$$

The relative fuzzy weights for all criteria are given in Table 8.

These fuzzy weights need to be defuzzified, meaning to be made into single, nonfuzzy numbers. The relative nonfuzzy weight of each criterion (*M*_i_) is calculated by taking the average of the fuzzy weight for each criterion, as in equation (6). Later on, using these single numbers *M*_*i*_'*s*, the normalized weights (*N*_i_) of each criterion are calculated using equation (7). The averaged and normalized weights are tabulated in Table 9.

It should be pointed out that these normalized weights of the criteria will be used as the criteria weights in the fuzzy ELECTRE method. In the following tables, the suppliers' comparison according to each seven criteria is done with the same methodology as before, using the pairwise comparisons with the inquiry form in Appendix C. First, the suppliers were compared in terms of quality. The results are given in Table 10.

Then, the geometric means for each supplier were calculated, and the fuzzy numbers were summed. From the obtained sums, the reverse values were calculated, and they were put in an increasing order as all triangular numbers should be. All these calculations for the quality criterion are shown in Table 11.

The relative fuzzy weights for quality are calculated and tabulated in Table 12.

After that, the fuzzy weights were normalized, and the results are given in Table 13.

Next comes the comparison of the suppliers according to the cost criterion, as given in Table 14.

Normalized weights of suppliers according to the cost criterion can be seen in Table 15.

Suppliers' comparison according to the delivery criterion can be seen in Table 16.

The suppliers were then compared in terms of service, and the results of those comparisons are given in Table 17.

According to the pollution control criterion, the suppliers' comparison is given in Table 18.

Their calculated geometric means, total and reverse values, and the increased order are given in Table 19.

Suppliers were also compared according to the green product criterion. The results are given in Table 20.

The last criterion according to which the suppliers were compared was the environment management criterion. The results are given in Table 21.

When all of the weights for the individual criteria are calculated and normalized, we put them all together, as tabulated in Table 22.

In the next step, the weights of the suppliers for each criterion are multiplied with the weights for the criteria. The results are given in Table 23.

So, the results for each supplier are given in Table 24.

The suppliers are arranged according to these results from the biggest to the smallest. The ranking is given in the following, where the order of significance would be 3-1-4-5-2.

### 4.3. Implementation of Fuzzy TOPSIS

Fuzzy TOPSIS is a modification of the TOPSIS method, using fuzzy numbers in order to escape the vagueness and uncertainty of human judgement. TOPSIS is a multiple-criteria decision-making method which helps in identification of solutions from a limited set of alternatives. TOPSIS depends on decision points' nearness to the ideal solution. So, in the fuzzy TOPSIS, an alternative that is nearest to the fuzzy positive ideal solution (FPIS) and farthest from the fuzzy negative ideal solution (FNIS) is chosen as the optimal. FPIS includes the best performance values for each alternative, while FNIS the worst ones.

The steps of the fuzzy TOPSIS were previously described, and how this method was implemented using the firm's data is given in the following.

According to the assessment in FTOPSIS (the forms given in Appendixes D and E) of the decision makers (the managers and staff), the fuzzy rating of the 12 decision makers for the 7 criteria is tabulated in Table 25. The comparison tables used in FAHP were created with the common decision of all decision makers. In this way, the weight of each criterion was obtained. We could use these weights in the FTOPSIS and FELECTRE methods. But differently, we took the fuzzy values of all decision makers about the criteria separately and obtained the arithmetic average of this. Since our aim is to find the final result with the common view of all of these methods, we aimed to find a better result by finding the weights in FTOPSIS and FELECTRE and supporting each other's decisions in different aspects.

The weights of the criteria are also calculated and tabulated in Table 26.

Criteria weights of FAHP can be used in FTOPSIS. But, different methods were used to solve the problem. We wanted to have a difference in these methods and have results obtained with different views and methods. Thus, we tried to catch dominance from different points while finding the dominant solution.

According to the alternative ratings by the decision makers, the aggregate fuzzy decision matrix is constructed, where the aggregate fuzzy weights $\tilde{w_{ij}}$ of each criterion are obtained according to equations (10) to (12). Using these results, a normalized fuzzy decision matrix is formed by using equation (14), where we should have in mind what the benefit and cost criteria are. In the next step, the weighted normalized fuzzy decision matrix $\bar{v}$ is constructed by multiplying the weights (${\tilde{w}}_{j}$) of the evaluation criteria with the normalized fuzzy decision matrix (${\tilde{r}}_{ij}$), as in equation (15). It is tabulated in Table 27.

(*A* ^*∗*^) and (*A* ^−^) of the alternatives are calculated as given in equation (17) and equation (18), respectively (Tables 28 and 29).

Next, the distances *d*_*i*_^*∗*^ and *d*_*i*_^−^ of each weighted alternative from the FPIS and the FNIS are calculated using equations (18) and (19), respectively. The calculations are given in the following tables. The calculations for distance *d*_*i*_^*∗*^ are given in Table 30.

Distance *d*_*i*_^−^ is calculated in the same manner but only using *A* ^−^. The calculations are given in Table 31. FNIS can be seen in Table 19. The sum of FNIS of the supplier can be seen in Table 32.

In the last step, the closeness coefficient *CC*_*i*_ of each alternative is calculated using equation (20), and the results are given in Table 33.

The alternative with the highest closeness coefficient is the best alternative (closest to the FPIS and furthest from the FNIS). The ranking is tabulated in Table 34.

### 4.4. Implementation of Fuzzy ELECTRE

The decision matrix that was constructed in fuzzy TOPSIS will be used also for fuzzy ELECTRE, but it will be transposed, as given in Table 35.

Next, this matrix is normalized using equation (22). For example,

(44) $$\begin{array}{c} \overset{˜}{n_{11}}=\frac{\overset{˜}{x_{11}}}{\sqrt{\sum_{i=1}^{m}{\left(s\left(\overset{˜}{x_{11}}|,|\;|0\right)\right)}^{2}}}=\frac{\left(1,4.83,9\right)\;}{\sqrt{{\left(\left(1+2*4.83+9\right)/4\right)}^{2}+{\left(\left(3+2*5.67+9\right)/4\right)}^{2}+{\left(\left(3+2*6.5+9\right)/4\right)}^{2}+{\left(\left(3+2*6.5+9\right)/4\right)}^{2}+{\left(\left(1+2*4+7\right)/4\right)}^{2}}}\\ =\frac{\left(1,\;4.83,\;9\right)}{12.3}=\left(0.08,\;0.39,\;0.73\right). \end{array}$$

The other elements are calculated in the same manner, and the normal fuzzy decision matrix is constructed, given in Table 36.

It was mentioned earlier that there are two ways to calculate the weighted fuzzy decision matrix, depending on whether the weights are single or fuzzy triangular numbers. In this case, we will apply the single number weights that we used in fuzzy AHP, given in Table 37.

Thus, calculated by equation (26), the weighted fuzzy decision matrix is obtained.

In the next step, the concordance and discordance sets are determined according to the relations in equations (27) and (28), respectively. These relations are using the equation for distance between two fuzzy numbers, where the second one is zero, like in equation (19). Therefore, this distance was calculated for each supplier according to each criteria. The concordance sets are used to constitute the fuzzy concordance matrix (${\tilde{I}}_{KL}$), whose elements are calculated with equation (29):

(45) $$\begin{array}{c} \tilde{I}=\left[\begin{array}{ccccccccccccccc} \ldots & \ldots & \ldots & 0,56 & 0,56 & 0,56 & 0,55 & 0,55 & 0,55 & 0,46 & 0,46 & 0,46 & 0,63 & 0,63 & 0,63\\ 0,44 & 0,44 & 0,44 & \ldots & \ldots & \ldots & 0,33 & 0,33 & 0,33 & 0,62 & 0,62 & 0,62 & 0,57 & 0,57 & 0,57\\ 0,45 & 0,45 & 0,45 & 0,67 & 0,67 & 0,67 & \ldots & \ldots & \ldots & 0,66 & 0,66 & 0,66 & 0,51 & 0,51 & 0,51\\ 0,54 & 0,54 & 0,54 & 0,38 & 0,38 & 0,38 & 0,34 & 0,34 & 0,34 & \ldots & \ldots & \ldots & 0,44 & 0,44 & 0,44\\ 0,37 & 0,37 & 0,37 & 0,43 & 0,43 & 0,43 & 0,49 & 0,49 & 0,49 & 0,56 & 0,56 & 0,56 & \ldots & \ldots & \ldots \end{array}\right]. \end{array}$$

Discordance sets and the elements of the weighted fuzzy decision matrix are used for constituting the fuzzy discordance matrix $\tilde{{\text{NI}}_{KL}}$. Its elements are calculated by equation (32) (Table 38).

The other elements are obtained in the same way, and an asymmetrical fuzzy discordance matrix $\tilde{\text{NI}}$ is constructed:

(46) $$\begin{array}{c} \tilde{N1}=\left[\begin{array}{ccccccccccccccc} \ldots & \ldots & \ldots & -1,02 & 0,141 & 1,016 & -1,42 & 0,592 & 2,132 & -2,13 & 0,296 & 2,132 & -0,91 & 0 & 0,906\\ -2,53 & 0,352 & 3,378 & \ldots & \ldots & \ldots & -4,23 & 0,881 & 6,341 & -1,63 & 0,339 & 2,438 & -1,69 & -0,35 & 1,267\\ -1,43 & 0 & 1,433 & -0,95 & 0,132 & 1,271 & \ldots & \ldots & \ldots & -12,5 & 0 & 12,51 & -0,37 & 0 & 0,492\\ -4,41 & 1,226 & 4,415 & -11,4 & 0 & 11,4 & -19,3 & 0 & 19,33 & \ldots & \ldots & \ldots & -2,18 & 0,303 & 2,179\\ -2,36 & -0,25 & 1,77 & -3,75 & 0 & 3,749 & -1,6 & 0 & 1,601 & -0,42 & 0,176 & 0,635 & \ldots & \ldots & \ldots \end{array}\right]. \end{array}$$

The effective concordance matrix (*F*) is constructed, composed of either 0 or 1, where 1 represents the priority of one choice over another:

(47) $$\begin{array}{c} F=\left|\begin{array}{ccccc} - & 1 & 1 & 0 & 1\\ 0 & - & 0 & 1 & 1\\ 0 & 1 & - & 1 & 1\\ 1 & 0 & 0 & - & 0\\ 0 & 0 & 0 & 1 & - \end{array}\right|. \end{array}$$

In the same manner, the effective discordance matrix (*G*) is constructed, using equation (38) for the threshold value $\tilde{\text{NĪ}}$. In addition, the privilege will be bigger if $\tilde{I_{KL}}\geq \tilde{\text{Ī}}\;\mathrm{and}\;S\left(\tilde{NI_{KL}}|,|\tilde{N\text{Ī}}\right)\leq 0$:

(48) $$\begin{array}{c} G=\left|\begin{array}{ccccc} - & 1 & 0 & 1 & 1\\ 0 & - & 0 & 0 & 1\\ 1 & 1 & - & 1 & 1\\ 0 & 1 & 1 & - & 1\\ 1 & 1 & 1 & 1 & - \end{array}\right|. \end{array}$$

In the end, the general matrix *H* is constructed by reciprocal multiplication of *f*_*ij*_ and *g*_*ij*_ elements (equation (40)):

(49) $$\begin{array}{c} H=\left|\begin{array}{ccccc} - & 1 & 0 & 0 & 1\\ 0 & - & 0 & 0 & 1\\ 0 & 0 & - & 1 & 1\\ 0 & 0 & 0 & - & 0\\ 0 & 0 & 0 & 1 & - \end{array}\right|. \end{array}$$

This matrix shows the relative privileges in choices, so the ranking would be as given in Table 39.

### 4.5. Implementation of Artificial Neural Network

**Figure 2:**
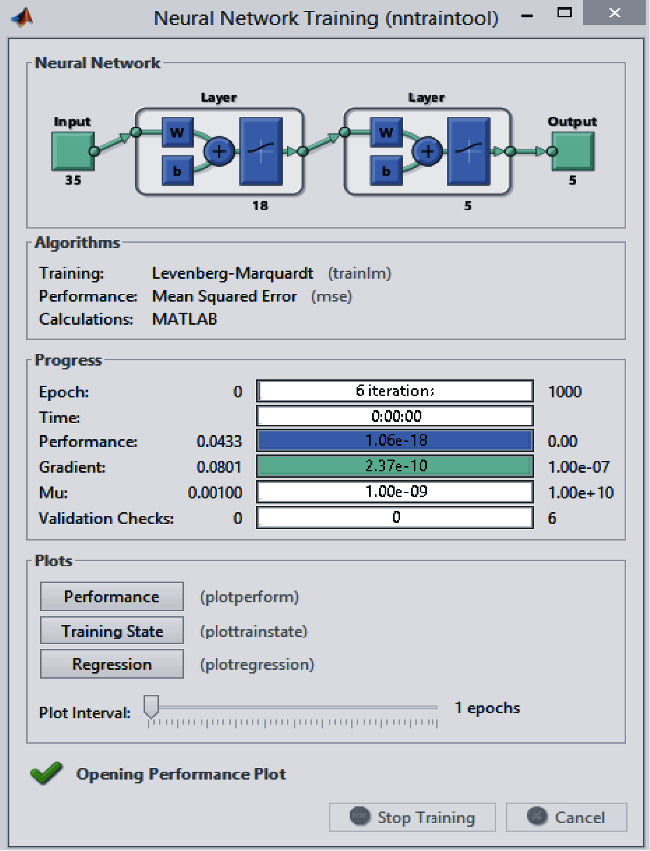
Neural network training output window.

For the implementation of ANN, the firm's data (each line of the comparison table) from the previous five years were set as inputs. On the other hand, as outputs were taken, the orders of the suppliers were according to the firm's net profit, from the supplier that brought the most money to the one that brought the least. MATLAB Toolbox was used to train the network. There were thirty-five neurons in the input layer for each five years, eighteen neurons in the hidden layer, and five neurons in the output layer. The network was trained using the multilayer feed-forward backpropagation algorithm to test its performance. The learning algorithm that was used was the Levenberg–Marquardt algorithm. The logarithmic sigmoid activation function was applied, and the learning rate was 0.1, while the momentum rate was 0.05.  Figure 2 represents the output window that is shown after the training of the neural network.

**Figure 3:**
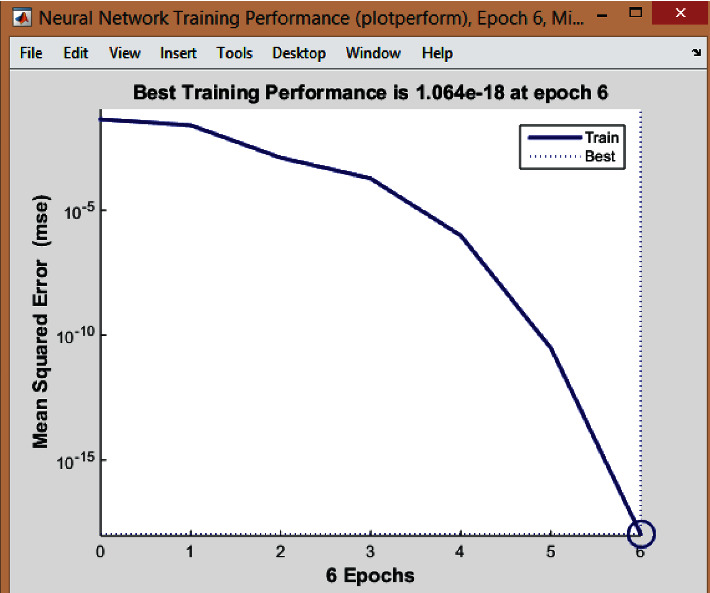
Neural network training performance.

The network is trained until a satisfying accuracy is obtained. Training automatically stops when generalization stops improving, as indicated by an increase in the mean square error (MSE) of the validation samples. The MSE is the average squared difference between outputs and targets. Lower values are better, while zero means no error. So, from Figure 3, it is observed that the best validation performance is 1.064^*e*−18^ at epoch 6, where the mean squared error is 0. The learning curve graph is shown in Figure 3.

## 5. Committee of Fuzzy MCDM and ANN to Select Green Supplier Selection

Some distinctions can be made between the fuzzy MCDM and ANN. Fuzzy logic is based on imprecise reasoning, and there is a limited data accuracy, so the decisions are made considering ambiguous, fuzzy, and raw data. On the other hand, ANN is based on the biological neural network composed of interconnected, concordant neurons whose aim is to yield outputs. It adjusts the data given to the system to those synaptic connections between the neurons, learns from the historic data on how the synapses work, and incorporates this in order to give more precise results. Here, it is worth mentioning that the prediction results are more promising because the data are trained until a minimum root mean square error is obtained. On the contrary, in the fuzzy methods, the root mean square error depends on the accuracy of construction of the fuzzy rules and how the data are prepared [61].

**Figure 4:**
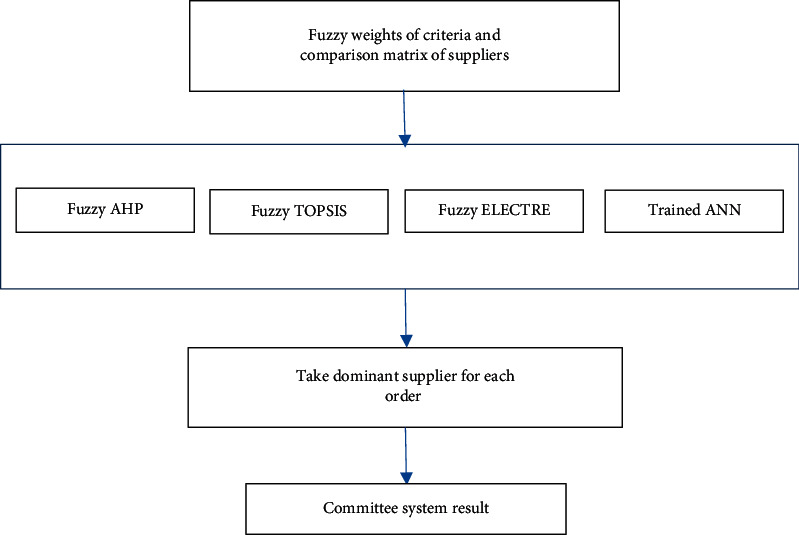
Structure of the proposed committee solution system.

In this study, firm's data from 2017 were analysed using fuzzy AHP, fuzzy TOPSIS, and fuzzy ELECTRE, and data from 2015 to 2017 were analysed with ANN in order to make adjustments to all the synaptic connections and help the network learn and give more precise results. To test the findings, the data set from 2018 was analysed with the fuzzy methods and ANN. The results that were obtained with the fuzzy MCDM methods and ANN are given in Table 40. Fuzzy MCDM and ANN give a decision altogether. There is a dominance order number for each method. For example, for supplier 4, the dominance supplier number is 5. FTOPSIS, FELECTRE, and ANN propose the same supplier number (5). The proposed solution system can be seen in Figure 4.

Obviously, there is a difference in the results. We should have in mind that for the construction of the neural network, the results from the net profit were considered as outputs. Consequently, the ANN gives better results because it is based on previous data of evaluations and net profits. Having said all this, it can be concluded that the ANN outperforms the fuzzy MCDM methods. It is better for forecasting with which of the suppliers long-term relationships can be formed because it is more reliable and easy to use, thus saving time and money. The ANN excludes the tiring and explicit decision-making process, and it deals better with the uncertainties and vagueness of the decisions [62].

ANN is designed according to the maximum profit criterion. ANN gives a sequence from the best profit to less profit. ANN gives a support to fuzzy MCDM models. It can be seen in Table 40 for supplier 2 that the result of FTOPSIS and FELECTRE is “1,” and ANN's result is “1.” ANN supports the result of the fuzzy MCDM models. For supplier 4, FTOPSIS, FELECTRE, and ANN give the same result “1.” For supplier 5, fuzzy MCDM models give different solutions, but ANN gives support to one of them. FELECTRE and ANN give the same result. ANN gives a support from the profit dimension for the given problem. The result of committee system: supplier 2, supplier 5, supplier 3, supplier 1, and supplier 2 is given. Also, it can be seen that the committee system result and the fuzzy ELECTRE result are the same.

## 6. Conclusion

The aim of this study was to define green supplier selection and raise awareness that companies should adopt this practice of choosing more ecofriendly suppliers. Firm's data were analysed according to the conventional and environmental criteria, and fuzzy MCDM methods and the artificial neural network were implemented. An efficient supplier selection process in one manufacturing system is crucial for a successful supply chain management. It improves productivity and reduces cost, thus satisfying the consumers' demands. More and more companies are now improving their SCM, but they forget to look from an environmental point of view. That is why this paper focuses on the application of some fuzzy multicriteria decision-making models, the FAHP, FTOPSIS, and FELECTRE, and the construction of ANN that will help the company to better and quickly find the right and ecofriendly supplier. In this study, there were five different suppliers which were compared according to seven different criteria: quality, cost, delivery, service, pollution control, green product, and environmental management. Fuzzy AHP, fuzzy TOPSIS, fuzzy ELECTRE, and ANN were applied using data from 2017. The given fuzzy MCDM techniques and ANN have been used as a committee. For the next studies, more fuzzy MCDM techniques and artificial intelligence methods can be used as a committee to obtain more useful decision.

## Figures and Tables

**Table 1:** Top ten criteria used in green supplier selection.

1	Environmental management systems
2	Quality
3	Price/cost
4	Service
5	Technology
6	Green design
7	Green image
8	Environmental performance
9	Environmental competences
10	Green collaboration with suppliers

**Table 2:** Frequency of the most important green criteria.

	Govindan et al.	Nielsen et al.
Duration	1996–2011	1996–2014
Number of reviewed papers	33	57
Criterion		
Environmental management system	11	20
Green image	4	8
Environmental competences	3	6
Design for environment	3	5
Environmental improvement costs	2	5
Environmental performance	3	5

**Table 3:** Linguistic terms and the corresponding triangular fuzzy numbers.

Saaty scale	Definition	Fuzzy triangular scale
1	Equally important	(1, 1, 1)
3	Weakly important	(2, 3, 4)
5	Fairly important	(4, 5, 6)
7	Strongly important	(6, 7, 8)
9	Absolutely important	(9, 9, 9)
2		(1, 2, 3)
4	The intermittent values between	(3, 4, 5)
6	Two adjacent scales	(5, 6, 7)
8		(7, 8, 9)

**Table 4:** Supplier selection criteria and the subcriteria for the firm.

Criteria	Subcriteria
Quality	Quality inspection methods
	Percentage of refused products
	High-quality employee
	Product performance

Cost	Discount dependent on purchasing quantity
	Lateness cost
	Holding cost

Delivery	Delivery speed
	Just-in-time delivery
	Transportation costs
	Flexibility on delivery time

Service	Stock management
	Responsiveness
	Design capability

Pollution control	Reduction of solid wastes
	Limited use of harmful materials
	Energy consumption

Green product	Green packaging
	Recycle
	Remanufacturing
	Reuse

Environmental management	Energy using product (EUP)
	Ozone depleting chemicals (ODC)
	Restriction of hazardous substance (RoHS)
	International Organization for Standardization (ISO 14001)
	Waste electrical electronic equipment (WEEE)

**Table 5:** Pairwise contribution matrix of the criteria.

	Quality	Cost	Delivery	Service	Pollution control	Green product	Environ. manag.
Quality	(1, 1, 1)	(1, 1, 1)	(4, 5, 4)	(1/2, 1/3, 1/4)	(2, 3, 4)	(4, 5, 6)	(1, 1, 1)
Cost	(1, 1, 1)	(1, 1, 1)	(2, 3, 4)	(2, 3, 4)	(2, 3, 4)	(4, 5, 6)	(1, 1, 1)
Delivery	(1/6, 1/5, 1/4)	(1/4, 1/3, 1/2)	(1, 1, 1)	(1/4, 1/3, 1/2)	(1/4, 1/3, 1/2)	(2, 3, 4)	(1/4, 1/3, 1/2)
Service	(1/4, 1/3, 1/2)	(1/4, 1/3, 1/2)	(2, 3, 4)	(1, 1, 1)	(1, 1, 1)	(2, 3, 4)	(1/4, 1/3, 1/2)
Pollution control	(1/4, 1/3, 1/2)	(1/4, 1/3, 1/2)	(2, 3, 4)	(1, 1, 1)	(1, 1, 1)	(2, 3, 4)	(1/4, 1/3, 1/2)
Green product	(1/6, 1/5, 1/4)	(1/6, 1/5, 1/4)	(1/4, 1/3, 1/2)	(1/4, 1/3, 1/2)	(1/4, 1/3, 1/2)	(1, 1, 1)	(1/6, 1/5, 1/4)
Environ. manag.	(1, 1, 1)	(1, 1, 1)	(2, 3, 4)	(2, 3, 4)	(2, 3, 4)	(4, 5, 6)	(1, 1, 1)

**Table 6:** Geometric means of the fuzzy comparison values.

Criteria	*r*_İ_
Quality	(1, 811, 2, 167, 2, 479)
Cost	(1, 640, 2, 167, 2, 339)
Delivery	(0, 387, 0, 496, 0, 672)
Service	(0, 672, 0, 854, 1, 104)
Pollution control	(0, 672, 0, 854, 1, 104)
Green product	(0, 256, 0, 313, 0, 410)
Environ. manag.	(1, 640, 2, 167, 2, 339)

**Table 7:** Geometric means, their total and reverse values, and the increasing order for criteria.

Criteria	$\tilde{r_{1}}$		
Quality	1,81144733	2,16783425	2,47939699
Cost	1,64067071	2,01527072	2,33986163
Delivery	0,38708428	0,49621125	0,6729501
Service	0,6729501	0,8547514	1,10408951
Pollution control	0,6729501	0,8547514	1,10408951
Green product	0,25614206	0,31330036	0,41016768
Environ. manag.	1,64067071	2,01527072	2,33986163
Total	7,08191529	8,7173901	10,450417
Reverse	0,14120474	0,11471323	0,09568996
Increasing order	0,09568996	0,11471323	0,14120474

**Table 8:** Relative fuzzy weights for each criterion.

Criteria	${\tilde{\omega }}_{i}$		
Quality	0,17333732	0,24867928	0,3501026
Cost	0,15699572	0,23117822	0,33039955
Delivery	0,03704008	0,056922	0,09502374
Service	0,06439457	0,0980513	0,15590267
Pollution control	0,06439457	0,0980513	0,15590267
Green product	0,02451022	0,0359397	0,05791762
Environ. manag.	0,15699572	0,23117822	0,33039955

**Table 9:** Averaged (*M*_*i*_) and normalized (*N*_*i*_) weights of criteria.

Criteria	*M*_*i*_	*N*_*i*_
Quality	0,25737307	0,2448594
Cost	0,2395245	0,22787864
Delivery	0,06299527	0,0599324
Service	0,10611618	0,10095673
Pollution control	0,10611618	0,10095673
Green product	0,03945585	0,03753747
Environ. manag.	0,2395245	0,22787864

**Table 10:** Suppliers' comparison according to the quality criterion.

	Supplier 1	Supplier 2	Supplier 3	Supplier 4	Supplier 5
Supplier 1	(1, 1, 1)	(1/4, 1/3, 1/2)	(1/6, 1/5, 1/4)	(1/6, 1/5, 1/4)	(2, 3, 4)
Supplier 2	(2, 3, 4)	(1, 1, 1)	(1/4, 1/3, 1/2)	(1/4, 1/3, 1/2)	(4, 5, 6)
Supplier 3	(4, 5, 6)	(2, 3, 4)	(1, 1, 1)	(1, 1, 1)	(6, 7, 8)
Supplier 4	(4, 5, 6)	(2, 3, 4)	(1, 1, 1)	(1, 1, 1)	(6, 7, 8)
Supplier 5	(1/4, 1/3, 1/2)	(1/6, 1/5, 1/4)	(1/8, 1/7, 1/6)	(1/8, 1/7, 1/6)	(1, 1, 1)

**Table 11:** Geometric means, their total and reverse values, and the increasing order for quality criterion.

Suppliers	${\overset{⌢}{r}}_{i}$		
Supplier 1	0,425142	0,525306	0,659754
Supplier 2	0,870551	1,107566	1,430969
Supplier 3	2,168944	2,536517	2,861938
Supplier 4	2,168944	2,536517	2,861938
Supplier 5	0,230527	0,267142	0,322197
Total	5,864106	6,973049	8,136796
Reverse	0,170529	0,143409	0,122898
Increasing order	0,122898	0,143409	0,170529

**Table 12:** Relative fuzzy weights of suppliers acc. to the quality criterion.

Suppliers	ῶ_i_		
Supplier 1	0,052249	0,075334	0,112507
Supplier 2	0,106989	0,158835	0,244022
Supplier 3	0,26656	0,36376	0,488043
Supplier 4	0,26656	0,36376	0,488043
Supplier 5	0,028331	0,038311	0,054944

**Table 13:** Averaged (*M*_*i*_) and normalized (*N*_*i*_) weights of suppliers acc. to the quality criterion.

Suppliers	*M*_i_	*N*_i_
Supplier 1	0,08003	0,077243
Supplier 2	0,169949	0,16403
Supplier 3	0,372788	0,359805
Supplier 4	0,372788	0,359805
Supplier 5	0,040529	0,039117

**Table 14:** Suppliers' comparison according to the cost criterion.

	Supplier 1	Supplier 2	Supplier 3	Supplier 4	Supplier 5
Supplier 1	(1, 1, 1)	(2, 3, 4)	(4, 5, 6)	(4, 5, 6)	(2, 3, 4)
Supplier 2	(1/4, 1/3, 1/2)	(1, 1, 1)	(2, 3, 4)	(2, 3, 4)	(1, 1, 1)
Supplier 3	(1/6, 1/5, 1/4)	(1/4, 1/3, 1/2)	(1, 1, 1)	(1, 1, 1)	(1/4, 1/3, 1/2)
Supplier 4	(1/6, 1/5, 1/4)	(1/4, 1/3, 1/2)	(1, 1, 1)	(1, 1, 1)	(1/4, 1/3, 1/2)
Supplier 5	(1/4, 1/3, 1/2)	(1, 1, 1)	(2, 3, 4)	(2, 3, 4)	(1, 1, 1)

**Table 15:** Averaged (*M*_*i*_) and normalized (*N*_*i*_) weights of suppliers according to the cost criterion.

Suppliers	*M*_*i*_	*N*_*i*_
Supplier 1	0,486238	0,45913
Supplier 2	0,207188	0,195637
Supplier 3	0,079214	0,074798
Supplier 4	0,079214	0,074798
Supplier 5	0,207188	0,195637

**Table 16:** Suppliers' comparison according to the delivery criterion.

	Supplier 1	Supplier 2	Supplier 3	Supplier 4	Supplier 5
Supplier 1	(1, 1, 1)	(1/6, 1/5, 1/4)	(1, 1, 1)	(1/4, 1/3, 1/2)	(2, 3, 4)
Supplier 2	(4, 5, 6)	(1, 1, 1)	(4, 5, 6)	(2, 3, 4)	(6, 7, 8)
Supplier 3	(1, 1, 1)	(1/6, 1/5, 1/4)	(1, 1, 1)	(1/4, 1/3, 1/2)	(2, 3, 4)
Supplier 4	(2, 3, 4)	(1/4, 1/3, 1/2)	(2, 3, 4)	(1, 1, 1)	(4, 5, 6)
Supplier 5	(1/4, 1/3, 1/2)	(1/8, 1/7, 1/6)	(1/4, 1/3, 1/2)	(1/6, 1/5, 1/4)	(1, 1, 1)

**Table 17:** Suppliers' comparison according to the service criterion.

	Supplier 1	Supplier 2	Supplier 3	Supplier 4	Supplier 5
Supplier 1	(1, 1, 1)	(2, 3, 4)	(1/6, 1/5, 1/4)	(1/4, 1/3, 1/2)	(2, 3, 4)
Supplier 2	(1/4, 1/3, 1/2)	(1, 1, 1)	(1/8, 1/7, 1/6)	(1/6, 1/5, 1/4)	(1, 1, 1)
Supplier 3	(4, 5, 6)	(6, 7, 8)	(1, 1, 1)	(2, 3, 4)	(6, 7, 8)
Supplier 4	(2, 3, 4)	(4, 5, 6)	(1/4, 1/3, 1/2)	(1, 1, 1)	(4, 5, 6)
Supplier 5	(1/4, 1/3, 1/2)	(1, 1, 1)	(1/8, 1/7, 1/6)	(1/6, 1/5, 1/4)	(1, 1, 1)

**Table 18:** Suppliers' comparison according to the pollution control criterion.

	Supplier 1	Supplier 2	Supplier 3	Supplier 4	Supplier 5
Supplier 1	(1, 1, 1)	(1/6, 1/5, 1/4)	(1/8, 1/7, 1/6)	(1/4, 1/3, 1/2)	(1/8, 1/7, 1/6)
Supplier 2	(4, 5, 6)	(1, 1, 1)	(1/4, 1/3, 1/2)	(2, 3, 4)	(1/4, 1/3, 1/2)
Supplier 3	(6, 7, 8)	(2, 3, 4)	(1, 1, 1)	(4, 5, 6)	(1, 1, 1)
Supplier 4	(2, 3, 4)	(1/4, 1/3, 1/2)	(1/6, 1/5, 1/4)	(1, 1, 1)	(1/6, 1/5, 1/4)
Supplier 5	(6, 7, 8)	(2, 3, 4)	(1, 1, 1)	(4, 5, 6)	(1, 1, 1)

**Table 19:** Distance measurement between two fuzzy numbers (*d*_v_) used for *d*_*i*_^*∗*^.

−8,44	−4,61	0	−7,33	−3,86	0	−7,33	−3,1	0	−7,33	−3,1	0	−8,44	−5,37	−2
−7,33	−3,02	0	−8	−3,7	0	−8	−4,38	0	−8	−4,38	0	−8	−3,7	0
−8,67	−5,75	0	−8	−4,79	0	−8,67	−5,75	0	−8	−5,27	0	−8,67	−6,23	−2
−8	−4,91	0	−8,67	−5,51	0	−7,33	−3,7	0	−8	−4,31	0	−8,67	−5,51	0
−8,67	−5,51	0	−8	−4,31	0	−7,33	−3,7	0	−8	−4,91	0	−7,33	−3,7	0
−6,67	−3,96	0	−6,67	−3,51	0	−6,89	−3,96	0	−6,44	−3,06	0	−6,67	−3,96	0
−7,33	−3,02	0	−7,33	−4,38	0	−7,33	−3,7	0	−7,33	−4,38	0	−6,33	−3,02	0

**Table 20:** Suppliers' comparison according to the green product criterion.

	Supplier 1	Supplier 2	Supplier 3	Supplier 4	Supplier 5
Supplier 1	(1, 1, 1)	(1/4, 1/3, 1/2)	(1, 1, 1)	(1/6, 1/5, 1/4)	(1, 1, 1)
Supplier 2	(2, 3, 4)	(1, 1, 1)	(2, 3, 4)	(1/4, 1/3, 1/2)	(2, 3, 4)
Supplier 3	(1, 1, 1)	(1/4, 1/3, 1/2)	(1, 1, 1)	(1/6, 1/5, 1/4)	(1, 1, 1)
Supplier 4	(4, 5, 6)	(2, 3, 4)	(4, 5, 6)	(1, 1, 1)	(4, 5, 6)
Supplier 5	(1, 1, 1)	(1/4, 1/3, 1/2)	(1, 1, 1)	(1/6, 1/5, 1/4)	(1, 1, 1)

**Table 21:** Suppliers' comparison according to the environment management criterion.

	Supplier 1	Supplier 2	Supplier 3	Supplier 4	Supplier 5
Supplier 1	(1, 1, 1)	(4, 5, 6)	(2, 3, 4)	(4, 5, 6)	(1, 1, 1)
Supplier 2	(1/6, 1/5, 1/4)	(1, 1, 1)	(1/4, 1/3, 1/2)	(1, 1, 1)	(1/6, 1/5, 1/4)
Supplier 3	(1/4, 1/3, 1/2)	(2, 3, 4)	(1, 1, 1)	(2, 3, 4)	(1/4, 1/3, 1/2)
Supplier 4	(1/6, 1/5, 1/4)	(1, 1, 1)	(1/4, 1/3, 1/2)	(1, 1, 1)	(1/6, 1/5, 1/4)
Supplier 5	(1, 1, 1)	(4, 5, 6)	(2, 3, 4)	(4, 5, 6)	(1, 1, 1)

**Table 22:** Matrix for fuzzy AHP.

Criteria	Weights	Supplier 1	Supplier 2	Supplier 3	Supplier 4	Supplier 5
Quality	0,244859397	0,07724287	0,16403006	0,35980493	0,35980493	0,0391172
Cost	0,227878636	0,45912954	0,19563728	0,07479795	0,07479795	0,19563728
Delivery	0,059932396	0,10444845	0,49544391	0,10444845	0,24889544	0,04676376
Service	0,100956731	0,12527306	0,05416529	0,50442192	0,26197443	0,05416529
Pollution control	0,100956731	0,0391172	0,16403006	0,35980493	0,07724287	0,35980493
Green product	0,037537474	0,08865565	0,23998168	0,08865565	0,49405136	0,08865565
Environ. manag.	0,227878636	0,35629843	0,06509837	0,1572064	0,06509837	0,35629843

**Table 23:** Multiplication of the weights of the criteria with the weights for each criterion.

Criteria	Supplier 1	Supplier 2	Supplier 3	Supplier 4	Supplier 5
Quality	0,018913643	0,040164303	0,088101618	0,088101618	0,009578215
Cost	0,104625813	0,044581556	0,017044855	0,017044855	0,044581556
Delivery	0,006259846	0,02969314	0,006259846	0,0149169	0,002802664
Service	0,012647158	0,005468351	0,050924788	0,026448082	0,005468351
Pollution control	0,003949145	0,016559939	0,036324729	0,007798188	0,036324729
Green product	0,003327909	0,009008306	0,003327909	0,01854544	0,003327909
Environ. manag.	0,0811928	0,014834528	0,03582398	0,014834528	0,0811928
Sum	0,230916315	0,160310124	0,237807726	0,187689611	0,183276224

**Table 24:** Results for each supplier for fuzzy AHP.

Supplier 1	0,230916

Supplier 2	0,16031
Supplier 3	0,237808
Supplier 4	0,18769
Supplier 5	0,183276

**Table 25:** The fuzzy ratings of the decision makers for the criteria.

	Quality			Cost			Delivery			Service			Pollution control			Green product			Environ. manag.		
DM 1	5	9	9	5	7	9	3	5	7	3	5	7	5	7	9	3	5	7	5	7	9
DM 2	5	9	9	7	9	9	3	5	7	5	7	9	5	7	9	1	3	5	7	9	9
DM 3	5	9	9	7	9	9	5	7	9	3	5	7	3	5	7	3	5	7	5	7	9
DM 4	5	7	9	3	5	7	3	5	7	5	7	9	3	5	7	3	5	7	5	7	9
DM 5	5	7	9	7	9	9	3	5	7	5	7	9	5	7	9	3	5	7	5	7	9
DM 6	5	7	9	3	5	7	3	5	7	3	5	7	3	5	7	3	5	7	5	7	9
DM 7	5	9	9	5	7	9	3	5	7	5	7	9	5	7	9	3	5	7	7	9	9
DM 8	5	9	9	5	7	9	3	5	7	5	7	9	5	7	9	3	5	7	5	7	9
DM 9	5	9	9	5	7	9	3	5	7	5	7	9	5	7	9	3	5	7	5	7	9
DM 10	5	9	9	7	9	9	3	5	7	5	7	9	5	7	9	3	5	7	5	7	9
DM 11	5	7	9	5	7	9	3	5	7	5	7	9	5	7	9	3	5	7	5	7	9
DM 12	5	7	9	5	7	9	3	5	7	5	7	9	5	7	9	3	5	7	5	7	9

**Table 26:** Weights of the criteria for FTOPSIS.

Weights	5 8, 17 9	3 7, 33 9	3 5, 17 9	3 6, 5 9	3 6, 5 9	1 4, 83 7	5 7, 33 9

**Table 27:** Weighted normalized fuzzy decision matrix.

	Supplier 1	Supplier 2	Supplier 3	Supplier 4	Supplier 5
Quality	0, 556 4, 386 9	1, 667 5, 142 9	1, 667 5, 898 9	1, 667 5, 142 9	0, 556 3, 63 9
Cost	1, 667 5, 975 9	1 5, 296 9	1 4, 617 9	1 4, 617 9	1 5, 296 9
Delivery	0, 333 3, 253 9	1 4, 21 9	0, 333 3, 253 9	1 3, 731 9	0, 333 2, 775 9
Service	1 4, 093 9	0, 333 3, 491 9	1, 667 5, 296 9	1 4, 694 9	0, 333 3, 491 9
Pollution control	0, 333 3, 253 9	1 4, 694 9	1, 667 5, 296 9	1 4, 093 9	1, 667 5, 296 9
Green product	0, 333 3, 043 7	0, 333 3, 491 7	0, 111 3, 043 7	0, 556 3, 938 7	0, 333 3, 043 7
Environment management	1, 667 5, 975 9	1, 667 4, 617 9	1, 667 5, 296 9	1, 667 4, 617 9	2, 778 5, 975 9

**Table 28:** *A* ^*∗*^.

9	9	9

9	9	9
9	9	9
9	9	9
9	9	9
7	7	7
9	9	9

**Table 29:** *A* ^−^.

0,56	0,56	0,56

1	1	1
0,33	0,33	0,33
0,33	0,33	0,33
0,33	0,33	0,33
0,11	0,11	0,11
1,67	1,67	1,67

**Table 30:** Distance measurement between two fuzzy numbers (*d*_v_) used for *d*_*i*_^−^.

0 3,8302 8,4444	1,1111 4,5864 8,4444	1,1111 5,3426 8,4444	1,1111 5,3426 8,4444	0 3,0741 6,4444

0,6667 4,9753 8	0 4,2963 8	0 3,6173 8	0 3,6173 8	0 4,2963 8
0 2,9198 8,6667	0,6667 3,8765 8,6667	0 5,9198 8,6667	0,6667 3,3981 8,6667	0 2,4414 6,6667
0,6667 3,7593 8,6667	0 3,1574 8,6667	1,3333 4,963 8,6667	0,6667 4,3611 8,6667	0 3,1574 8,6667
0 3,1574 8,6667	0,6667 4,3611 8,6667	1,3333 4,963 8,6667	0,6667 3,7593 8,6667	1,3333 4,963 8,6667
0,2222 2,9321 6,8889	−6,6667 −3,509 0	−6,889 −3,957 0	−6,444 −3,062 0	−6,667 −3,957 0
0 4,3086 7,3333	0 2,6506 7,3333	0 3,6296 7,3333	0 3,9506 7,3333	1,1111 4,3086 7,3333

**Table 31:** The FNIS (square root of the average).

	FNIS supplier 1	FNIS supplier 2	FNIS supplier 3	FNIS supplier 4	FNIS supplier 5
Quality	5,35348589	5,58505315	5,80478267	5,80478267	4,12232926
Cost	5,45277118	5,24271437	5,06901513	5,06901513	5,24271437
Delivery	5,28002744	5,4949414	5,28002744	5,38835059	4,09897034
Service	5,46771085	5,32542119	5,81721268	5,61470823	5,32542119
Pollution control	5,32542119	5,61470823	5,81721268	5,46771085	5,81721268
Green product	4,32447978	4,34968754	4,58668283	4,11926551	4,47588474
Environ. manag.	4,91060666	4,56376745	4,72412211	4,56376745	4,95233081

**Table 32:** *d*_*i*_^−^ (the sum of FNIS of the supplier).

	FNIS Supplier 1	FNIS Supplier 2	FNIS Supplier 3	FNIS Supplier 4	FNIS Supplier 5
Sum (*d*_*i*_^−^)	36,114503	36,1762933	37,0990555	36,0276004	34,0348634

**Table 33:** The closeness coefficient to the positive and negative ideal solutions.

Supplier 1	0,49705148

Supplier 2	0,50321685
Supplier 3	0,51682306
Supplier 4	0,50645299
Supplier 5	0,48326187

**Table 34:** Ranking of the suppliers according to fuzzy TOPSIS.

Supplier 3	0,51682306

Supplier 4	0,50645299
Supplier 2	0,50321685
Supplier 1	0,49705148
Supplier 5	0,48326187

**Table 35:** Aggregate fuzzy decision matrix (transposed).

	Quality			Cost			Delivery			Service			Pollution control			Green product			Environ. manag.		
Supplier 1	1	4,83	9	5	7,33	9	1	5,67	9	3	5,67	9	1	4,83	9	3	5,667	9	3	7,33	9
Supplier 2	3	5,67	9	3	6,5	9	3	7,33	9	1	4,83	9	3	6,5	9	3	6,5	9	3	5,67	9
Supplier 3	3	6,5	9	3	5,67	9	1	5,67	9	5	7,33	9	5	7,33	9	1	5,667	9	3	6,5	9
Supplier 4	3	6,5	9	3	5,67	9	3	6,5	9	3	6,5	9	3	5,67	9	5	7,333	9	3	5,67	9
Supplier 5	1	4	7	3	6,5	9	1	4,83	7	1	4,83	9	5	7,33	9	3	5,667	9	5	7,33	9

**Table 36:** Normal fuzzy decision matrix.

	Quality	Cost	Delivery	Service	Pollution control	Green product	Environ. manag.
Supplier 1	0,08 0,39 0,73	0,36 0,52 0,64	0,08 0,45 0,71	0,23 0,43 0,68	0,07 0,34 0,64	0,22 0,414 0,68	0,26 0,63 0,77
Supplier 2	0,24 0,46 0,73	0,21 0,46 0,64	0,24 0,58 0,71	0,08 0,37 0,68	0,21 0,46 0,64	0,22 0,475 0,66	0,26 0,48 0,77
Supplier 3	0,24 0,53 0,73	0,21 0,4 0,64	0,08 0,45 0,71	0,38 0,56 0,68	0,35 0,52 0,64	0,07 0,414 0,66	0,26 0,56 0,77
Supplier 4	0,24 0,53 0,73	0,21 0,4 0,64	0,24 0,51 0,71	0,23 0,49 0,68	0,21 0,4 0,64	0,37 0,536 0,66	0,26 0,48 0,77
Supplier 5	0,08 0,32 0,57	0,21 0,46 0,64	0,08 0,38 0,71	0,08 0,37 0,68	0,35 0,52 0,64	0,22 0,414 0,66	0,43 0,63 0,77

**Table 37:** Weights of criteria according to FAHP.

Weights	0,244859397	0,227878636	0,059932396	0,100956731	0,100956731	0,037537474	0,227878636

**Table 38:** *k* = 1 and *L* = 2.

s(v11, v21)	−0,01819

s(v12, v22)	0,014862
s(v13, v23)	−0,00632
s(v14, v24)	0,00704
s(v15, v25)	−0,00952
s(v16, v26)	−0,00114
s(v17, v27)	0,01629

**Table 39:** Ranking of the suppliers according to fuzzy ELECTRE.

Supplier 3	0,51682306

Supplier 4	0,50645299
Supplier 2	0,50321685
Supplier 1	0,49705148
Supplier 5	0,48326187

**Table 40:** Committee of fuzzy MCDM and ANN results.

Suppliers	FAHP order	FTOPSIS order	FELECTRE order	ANN order	Committee system result
Supplier 1	1	4	4	3	4
Supplier 2	2	1	1	1	1
Supplier 3	3	2	3	4	3
Supplier 4	4	5	5	5	5
Supplier 5	5	3	2	2	2

**Table 41:** Questionnaire about the importance of each criterion.

Traditional criteria	Not at all important	Slightly important	Moderately important	Very important	Extremely important
Price	☐ 1	☐ 2	☐ 3	☐ 4	☐ 5
Quality	☐ 1	☐ 2	☐ 3	☐ 4	☐ 5
Delivery time	☐ 1	☐ 2	☐ 3	☐ 4	☐ 5
Warranties & obligations	☐ 1	☐ 2	☐ 3	☐ 4	☐ 5
Payment terms	☐ 1	☐ 2	☐ 3	☐ 4	☐ 5
Risk factor	☐ 1	☐ 2	☐ 3	☐ 4	☐ 5
Position in the sector	☐ 1	☐ 2	☐ 3	☐ 4	☐ 5
Technology	☐ 1	☐ 2	☐ 3	☐ 4	☐ 5
Geographical location	☐ 1	☐ 2	☐ 3	☐ 4	☐ 5
Service	☐ 1	☐ 2	☐ 3	☐ 4	☐ 5
Flexibility	☐ 1	☐ 2	☐ 3	☐ 4	☐ 5
Just-in-time delivery	☐ 1	☐ 2	☐ 3	☐ 4	☐ 5
Minimum order quantity	☐ 1	☐ 2	☐ 3	☐ 4	☐ 5
Technical capacity	☐ 1	☐ 2	☐ 3	☐ 4	☐ 5
Production capacity	☐ 1	☐ 2	☐ 3	☐ 4	☐ 5
Experience	☐ 1	☐ 2	☐ 3	☐ 4	☐ 5
Problem solving ability	☐ 1	☐ 2	☐ 3	☐ 4	☐ 5
Environmental criteria	☐ 1	☐ 2	☐ 3	☐ 4	☐ 5
Ecological materials	☐ 1	☐ 2	☐ 3	☐ 4	☐ 5
Green product	☐ 1	☐ 2	☐ 3	☐ 4	☐ 5
Pollution control	☐ 1	☐ 2	☐ 3	☐ 4	☐ 5
Green image	☐ 1	☐ 2	☐ 3	☐ 4	☐ 5
Green competencies	☐ 1	☐ 2	☐ 3	☐ 4	☐ 5
Environment management	☐ 1	☐ 2	☐ 3	☐ 4	☐ 5
Green purchasing	☐ 1	☐ 2	☐ 3	☐ 4	☐ 5
Life cycle assessment	☐ 1	☐ 2	☐ 3	☐ 4	☐ 5
R&D green products	☐ 1	☐ 2	☐ 3	☐ 4	☐ 5
Corporate social responsibility	☐ 1	☐ 2	☐ 3	☐ 4	☐ 5
Green innovation	☐ 1	☐ 2	☐ 3	☐ 4	☐ 5
Hazardous substance management	☐ 1	☐ 2	☐ 3	☐ 4	☐ 5
Environment protection	☐ 1	☐ 2	☐ 3	☐ 4	☐ 5
Green design	☐ 1	☐ 2	☐ 3	☐ 4	☐ 5
Availability of “clean” technologies	☐ 1	☐ 2	☐ 3	☐ 4	☐ 5

**Table 42 tab42:** Inquiry form for evaluation of criteria.

Abs. imp. (9, 9, 9)	Str. imp. (6, 7, 8)	Fair. imp. (4, 5, 6)	Weak. imp. (2, 3, 4)	Criterion	Eq. imp. (1, 1, 1)	Criterion	Weak. imp. (2, 3, 4)	Fair. imp. (4, 5, 6)	Str. imp. (6, 7, 8)	Abs. imp. (9, 9, 9)
				Quality		Cost				
				Quality		Delivery				
				Quality		Service				
				Quality		Pollution control				
				Quality		Green product				
				Quality		Environ. manag.				
				Cost		Delivery				
				Cost		Service				
				Cost		Pollution control				
				Cost		Green product				
				Cost		Environ. manag.				
				Delivery		Service				
				Delivery		Pollution control				
				Delivery		Green product				
				Delivery		Environ. manag.				
				Service		Pollution control				
				Service		Green product				
				Service		Environ. manag.				
				Pollution control		Green product				
				Pollution control		Environ. manag.				
				Green product		Environ. manag.				

^*∗*^The criteria are the seven criteria chosen as being the most important according to the survey in Appendix A.

**Table 43 tab43:** Inquiry form for evaluation of suppliers according to each criterion.

Abs. imp. (9, 9, 9)	Str. imp. (6, 7, 8)	Fair. imp. (4, 5, 6)	Weak. imp. (2, 3, 4)	Supplier	Eq. imp. (1, 1, 1)	Supplier	Weak. imp. (2, 3, 4)	Fair. imp. (4, 5, 6)	Str. imp. (6, 7, 8)	Abs. imp. (9, 9, 9)
				s1		s2				
				s1		s3				
				s1		s4				
				s1		s5				
				s2		s3				
				s2		s4				
				s2		s5				
				s3		s4				
				s3		s5				
				s4		s5				

^*∗*^The suppliers were given by name so that the staff know which of the suppliers they are giving points to.

**Table 44 tab44:** Form for assessing criteria.

	Very low importance	Low importance	Medium importance	High importance	Very high importance
Quality					
Cost					
Delivery					
Service					
Pollution control					
Green product					
Environ. manag.					

^*∗*^The criteria are the seven criteria chosen as being the most important according to the survey in Appendix A.

**Table 45 tab45:** Table of assessment values.

Fuzzy number	Assessment
(1, 1, 3)	Very poor (VP)
(1, 3, 5)	Poor (P)
(3, 5, 7)	Fair (F)
(5, 7, 9)	Good (G)
(7, 9, 9)	Very good (VG)

**Table 46:** Form for assessing suppliers in terms of each criterion.

	Supplier 1	Supplier 2	Supplier 3	Supplier 4	Supplier 5
Quality					
Cost					
Delivery					
Service					
Pollution control					
Green product					
Environ. manag.					

^*∗*^The suppliers were given by name so that the staff know which of the suppliers they are giving points to. ^*∗∗*^The criteria are the seven criteria chosen as being the most important according to the survey in Appendix A.

## Data Availability

All of the data are included in the manuscript in tables.

## Appendices

### Appendix A

According to you, for twill textile supply, what are the most things that a supplier should possess? Please assess the following TRADITIONAL and ENVIRONMENTAL criteria separately according to their importance.(Tables 41 [Table tab42] [Table tab43] [Table tab44] [Table tab45]–46)

### B An Inquiry Form for Evaluation of Criteria

Compare the criteria^*∗*^ by checking the cell of the criterion you think it has more advantage over the other.

### C An Inquiry Form for Evaluation of Suppliers according to Each Criterion

Compare the suppliers^*∗*^ by checking the cell of the supplier you think it has more advantage over the other.

### D

Please assess the criteria^*∗*^ according to their importance by checking the appropriate cell.

### E

Using the abbreviations given in the assessment column, please assess the suppliers^*∗*^ according to the given criteria^*∗∗*^.
